# Functionalized metal-organic frameworks as smart nanoplatforms: advancing tumor precision therapy through post-synthetic surface tailoring, challenges, and prospects for clinical translation

**DOI:** 10.1186/s12951-026-04757-9

**Published:** 2026-07-27

**Authors:** Shiva Velayati, Masoumeh Zahmatkeshan, Fariba Esmaeili, Seyed Mohammad Hosseini, Seyed Mahdi Rezayat, Mahmoud Reza Jaafari, Kazem Mousavizadeh, Gholamreza Taheripak, Moein Adel, Anna Lesniak, Iraj Alipourfard

**Affiliations:** 1 Cellular and Molecular Research Center, Iran University of Medical Sciences, Tehran, Iran; 2 Student Research Committee, Iran University of Medical Sciences, Tehran, Iran; 3 Department of Medical Nanotechnology, Faculty of Advanced Technologies in Medicine, Iran University of Medical Sciences, Tehran, Iran; 4 Research and Development Department, Nano Asayesh Parsina Co (NAP), Tehran, Iran; 5 Department of Medical Nanotechnology, School of Advanced Technologies in Medicine, Tehran University of Medical Sciences, Tehran, Iran; 6 Department of Pharmacology, School of Medicine, Tehran University of Medical Sciences, Tehran, Iran; 7 Nanotechnology Research Center, Pharmaceutical Technology Institute, Mashhad University of Medical Sciences, Mashhad, Iran; 8 Department of Pharmaceutical Nanotechnology, School of Pharmacy, Mashhad University of Medical Sciences, Mashhad, Iran; 9 Department of Pharmacology, School of Medicine, Iran University of Medical Sciences, Tehran, Iran; 10 Department of Biochemistry, School of Medicine, Iran University of Medical Sciences, Tehran, Iran; 11 Colorectal Research Center, Burn Research Center, Neuroscience Research Center, and Razi Drug Research Center, Iran University of Medical Sciences, Tehran, Iran; 12 Department of Pharmacotherapy and Pharmaceutical Care, Faculty of Pharmacy, Medical University of Warsaw, Banacha 1, Warsaw, 02-097 Poland

**Keywords:** Post-synthetic modifications (PSMs), Metal-organic frameworks (MOFs), Nanoplatforms, Cancer nanotherapy, Nanomedicine

## Abstract

Metal–organic frameworks (MOFs) have garnered significant attention as versatile nanocarriers for oncological drug delivery, owing to their exceptional porosity, structural tunability, extensive specific surface areas, and favorable drug loading and release kinetics, which collectively enable targeted payload delivery and reduced systemic toxicity. Nevertheless, the clinical promise of unmodified MOFs is severely curtailed by substantial in vivo biological barriers—including rapid bio-clearance, the immunosuppressive and acidic tumor microenvironment (TME), and inefficient cellular internalization—challenges that are ubiquitous across nanomedicine platforms and critically undermine therapeutic outcomes. This review provides a comprehensive examination of post-synthetic modification (PSM) as an indispensable strategy to surmount these obstacles. It systematically categorizes the diverse array of surface modifiers reported to date into natural polymers, synthetic polymers, nonpolymeric small molecules, inorganic materials, and multifunctional hybrid architectures, while elucidating how each class can enhance biocompatibility, colloidal stability, circulation half-life, active targeting, and stimuli-responsive cargo release. Crucially, the review critically appraises the intrinsic trade-offs introduced by surface engineering, including increased hydrodynamic diameter, impaired tumor penetration, heightened synthetic and characterization complexity, and unpredictable perturbations to biodegradation pathways and immune system interactions. Despite robust preclinical validation, the translational trajectory of surface-engineered MOF-based therapeutics remains encumbered by formidable challenges spanning scalable and reproducible manufacturing, long-term biosafety assessment, and stringent regulatory approval. By delineating current advances, critical limitations, and prospective directions, this review highlights the delicate equilibrium between surface functionalization and biological performance that must be achieved to realize the clinical potential of PSM-optimized MOF nanomedicines.

**Graphical abstract:**

**Figure:**
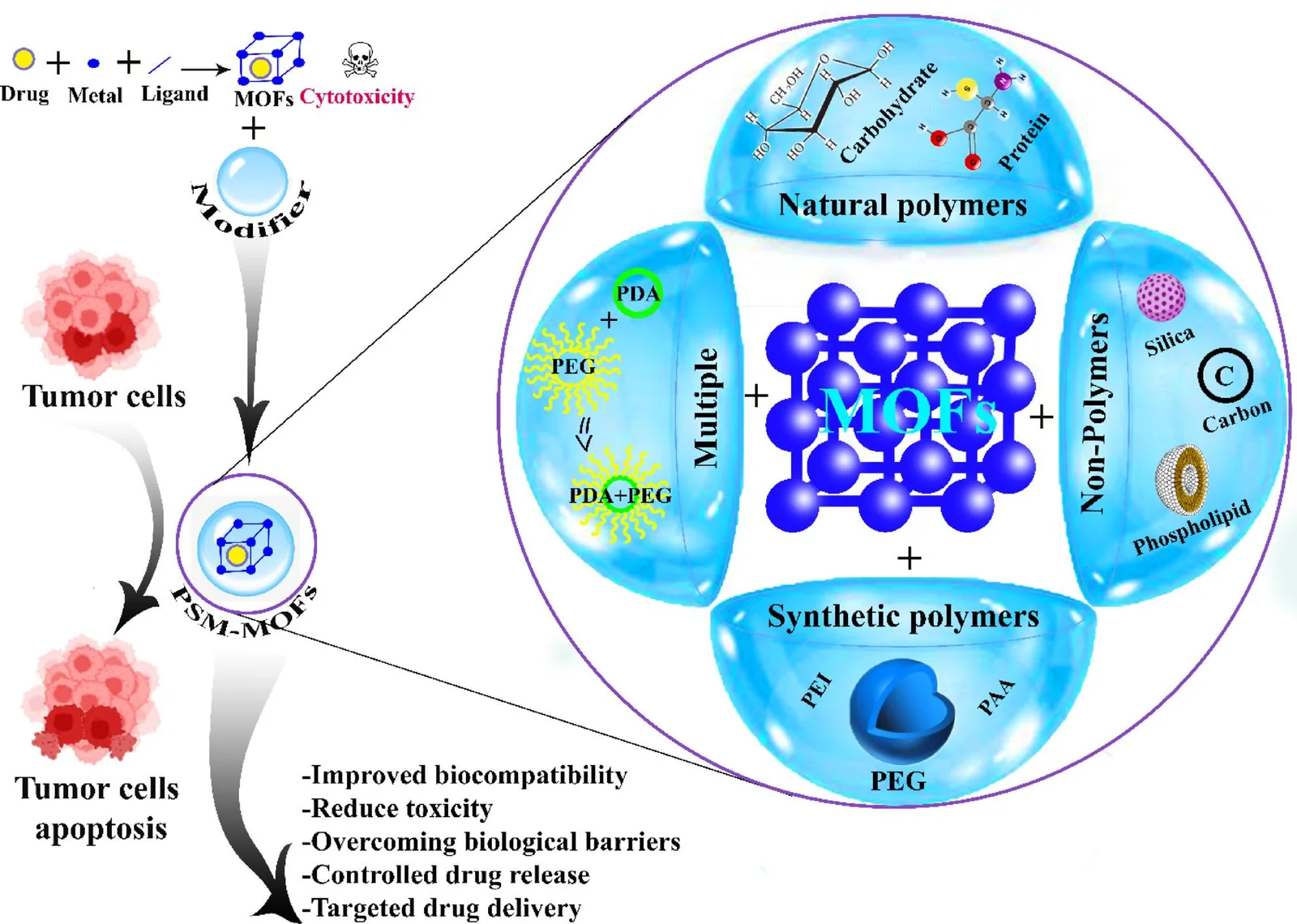

**Supplementary Information:**

The online version contains supplementary material available at https://doi.org/10.1186/s12951-026-04757-9.

## Highlights

MOFs, due to their tunable and porous structures, are used in tumor treatment as DDSs to improve drug targeting and reduce side effects.

Hybridizing polymer and biomimetic coatings with MOFs reduces immune system recognition, controls the release of drugs, and increases bioavailability.

MOFs’ prospects and approaching challenges in tumor treatment.

**Supplementary Information:**

The online version contains supplementary material available at https://doi.org/10.1186/s12951-026-04757-9.

## Introduction

Porous materials are attracting increasing attention and have become indispensable for biomedical applications due to their advantageous intrinsic characteristics, such as large surface area, high pore volume, and tunable pore dimensions. Extensive research has been directed toward the development of metal–organic frameworks (MOFs), also referred to as coordination polymers or porous coordination networks, which constitute an emerging and highly promising class of porous materials [1]. MOFs are crystalline solids generated through the self-assembly of metal nodes and organic linkers [2]. Owing to their highly tunable nature and mild synthesis conditions, MOFs allow for the rational structural design of numerous nanoscale metal–organic frameworks (NMOFs) and the incorporation of a wide range of functionalities, including targeted therapeutic biomolecules [3]. Compared with conventional porous materials (e.g., inorganic zeolites, silica-based nanomaterials, and organic nanocarriers like lipids or polymers), MOFs offer several advantages, including high surface area, tunable pore size and shape, high loading capacity, adjustable composition and structure, and extensive functional versatility [4]. Various methods have been employed for the synthesis of MOFs, including permeation, hydrothermal and solvothermal techniques, microwave-assisted synthesis, sonochemical and electrochemical processes, as well as one-pot approaches [1].

**Scheme 1:**
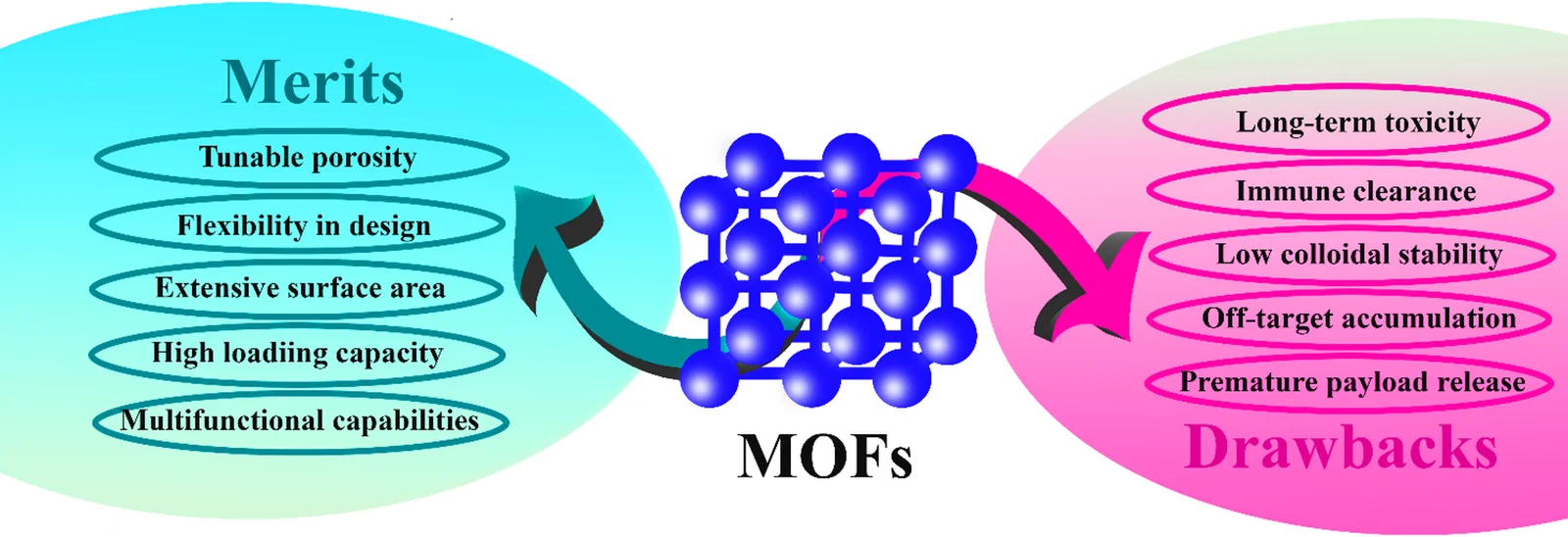
Merits and drawbacks of MOFs

Although research on MOFs for biomedical applications has progressed substantially, several challenges continue to hinder their widespread implementation, including long-term cytotoxicity, low colloidal stability, Off-target accumulation, immune clearance, and premature payload release [5] **(**Scheme 1**).** The versatility of MOFs is further enhanced by surface functionalization, which not only augments their dispersibility and targeting efficiency but also addresses the biological barriers that traditionally impede nanoparticle (NPs)-based drug delivery [6]. These barriers include the adsorption of bioproteins, which can mask the NPs and prompt immune recognition and the complex interplay within the tumor microenvironment (TME), which affects NPs penetration and efficacy [7]. Additionally, the cell membrane prevents NPs from being internalized into the cell, and drugs released within the cell are removed by efflux pumps, impeding their therapeutic effect [8].

Surface functionalization and modification strategies have emerged as effective solutions to address these limitations. Consequently, it is essential to modify synthesized MOFs to impart tailored physicochemical and biological properties [9, 10]. In the context of MOF crystals, two primary strategies exist for surface functionalization: in situ functionalization, which introduces functional groups during crystal synthesis, and post-synthetic modification (PSM), which occurs after crystal formation [11]. Although in situ functionalization enables control over the terminating crystal facet, size, and surface coverage, it is not addressed in the present paper [12]. The first reports of PSM for functionalizing internal pore surfaces appeared in 1999 [13] ; analogous PSM strategies can be adapted to selectively modify the external crystal surface, which constitutes the central focus of this review. PSMs are commonly applied to achieve sustained drug release, colloidal stability, long-term circulation, and the ability to overcome biological barriers. Based on recent literature, new perspectives and strategies for overcoming these obstacles are proposed, leveraging the multifunctional capabilities of MOFs to enhance therapeutic payload delivery and targeting precision [14].

This work examines recent developments in the outer-surface functionalization of MOF crystals using coating materials such as natural polymers, synthetic polymers, non-polymeric materials, and multilayer surface modifications based on various interactions, and explores their potential applications. The selection of appropriate functional materials enables the engineering of NPs with improved dispersibility and targeting capabilities. Additionally, the biocompatibility, metabolic fate, and regulatory considerations of PSM-functionalized MOFs are determined.

## Nanoscale metal–organic frameworks (NMOFs) and their stability

MOFs are hybrid porous coordination solids comprising inorganic metal-containing nodes and multitopic organic linkers [15], offering exceptional design flexibility [16]. Their functional pore walls, high specific surface area, and multiple coordination sites enable extensive functionalization and efficient site-specific drug delivery via host–guest interactions [17]. Atomic-level localization of inorganic and organic moieties within micro- or mesopores allows unparalleled tuning of electrostatic and dispersive forces governing these interactions [18]. Thermodynamic stability is determined by coordination bond strength between metal nodes and linkers, as predicted by Pearson’s hard and soft acids and bases (HSAB) principle [19, 20]. Stable MOFs result from pairing hard Lewis bases (e.g., carboxylate ligands) with high-valent metal ions (e.g., Ti^4+^, Zr^4+^, Al^3+^, Fe^3+^, Cr^3+^) or soft bases (e.g., imidazolate, pyrazolate, triazolate, tetrazolate ligands) with soft divalent ions (e.g., Zn^2+^, Cu^2+^, Mn^2+^) [21]. In biological environments, competing ions such as phosphate in phosphate-buffered saline (PBS) can displace organic ligands, reducing stability particularly for carboxylate-based MOFs with high-valent ions [22]. Thus, understanding biological context is essential for rational MOF design.

## Biological fate and design principles of biocompatible uncoated NMOFs

For clinical translation of NMOFs in tumor precision therapy, comprehensive assessment of in vivo biological fate and long-term safety is required beyond therapeutic efficacy [23]. Degradation, initiated by coordination bond hydrolysis and accelerated by acidic (tumor or lysosomal) pH, leads to metal ion leaching and linker liberation [24]. Toxic metal ions (e.g., Cr^3+^, Cu^2+^) may catalyze Fenton-type reactive oxygen species (ROS) generation, whereas biocompatible metals (Fe, Mg, Ca, Zn) are preferred [25]. Redox-active nodes (Fe, Cu, Mn) can also generate ROS exploited in photodynamic and chemodynamic therapy [26, 27]. A critical gap exists in long-term identification and quantification of degradation by-products. Intravenously administered MOFs are sequestered by the mononuclear phagocyte system (MPS), accumulating in liver and spleen [28]. Renal clearance is restricted to ultrasmall MOFs (< 5.5–6 nm), whereas therapeutic MOFs (50–200 nm) undergo slow, incomplete hepatobiliary excretion, with > 50% persisting for weeks [29], potentially causing granuloma, sinusoidal dilatation, necrosis, inflammation, and, for non-biodegradable MOFs (e.g., UiO-66), fibrosis and splenomegaly. Absence of standardized long-term large-animal studies constitutes a regulatory gap [30]. Rational design should prioritize intrinsic biodegradability and controlled clearance using essential metal ions (Fe, Zn, Ca, Mg) while avoiding toxic metals (Cr, As, Cd, Pb, Co) [23].

For biomedical MOFs, organic ligands must exhibit low toxicity and biocompatibility. Exogenous aromatic carboxylates (e.g., terephthalic acid [31] and trimesic acid [32]) risk renal, immunogenic, or endocrine toxicity, whereas endogenous ligands (fumarate [33], adenine [34], cyclodextrin (CD) [35], amino acids [36, 37]) are naturally metabolized and preferred. Unreacted modulators and residual solvents (e.g., DMF) further contribute to hepatotoxicity. Nucleobase, amino-acid-derived [38], and CD-based linkers degrade into nontoxic products (purines, oligopeptides, sugars, salts). When aromatic polycarboxylates are unavoidable, rapid clearance, enzyme-labile linkages (ester, disulfide) [39], or defect-rich structures are required [40]. The overall degradation product cocktail—not isolated components—determines biological fate [41]. Adherence to endogenous building blocks, labile coordination chemistry, and clearance-optimized size control enables MOFs that are sufficiently stable for therapy yet fully biodegradable [42].

NPs size, charge, and morphology govern systemic circulation and MPS evasion [43]. Size dictates enhanced permeability and retention (EPR) effect utilization: ultrasmall MOFs (< 6 nm) enable rapid renal clearance [44], whereas larger MOFs (30–100 nm) facilitate passive tumor accumulation [45]. Active targeting via receptor–ligand interactions is feasible [46]. Surface charge influences endocytic uptake and immunogenicity—positive charge enhances internalization but hydrophobic or strongly positive surfaces increase inflammatory risk [47, 48]. Morphology modulates clearance, with nanorods and nano-octahedra (aspect ratio < 2) eliminated faster than spherical particles due to reduced macrophage recognition [45, 49].

Surface chemistry critically governs MOF biological fate by mediating interactions with the biological environment and dictating barrier traversal capability [50, 51]. Uncoated MOFs, despite tunability and biodegradability, face significant limitations in overcoming sequential biological barriers (from injection to cellular uptake, trafficking, and efficacy) and remain vulnerable to efflux pump‑mediated drug resistance [8]. Thus, rational engineering of MOFs is essential to address these challenges.

## Biological barriers in cancer therapy

Upon systemic administration, NMOFs circulate within the bloodstream and interact extensively with various bioproteins. The adsorption of these bioproteins onto the NPs surface can impair the particles’ intended functional properties. Of particular concern is the binding of immune-related proteins, which renders the NPs susceptible to immunological recognition [43]. This recognition triggers their clearance from circulation or filtration by specialized organs. Consequently, foreign substances such as NMOFs are eliminated by the reticuloendothelial system (RES). Therefore, achieving effective cancer therapy requires maintaining a biodistribution profile that enables the NPs to reach the target tumor while evading detection by the body’s clearance mechanisms. The RES operates primarily in filtration and immune organs, including the liver and lymph nodes. Within these organs, the MPS [43] mediates clearance through immune cells such as monocytes and macrophages [52]. The TME comprises a dynamic network of cellular, molecular, and structural elements, including stromal cells, immune cells, blood vessels, and the extracellular matrix, which collectively influence tumor development, progression, and treatment response [53]. Attributes of the TME, including low pH, elevated glutathione (GSH) levels, and hypoxic conditions, present potential avenues for therapeutic approaches like immunomodulation, anti-angiogenesis, and interference with stromal interactions [7]. For successful cellular entry, NPs must traverse the cell membrane, whose physicochemical properties govern permeability. Although transport may be enhanced through binding to specific membrane receptors, discrepancies in size, shape, surface charge, or polarity, along with exocytosis and membrane push-back mechanisms, may obstruct efficient delivery. Consequently, the reciprocal relationship between NPs attributes and cell membrane characteristics ultimately determines delivery efficiency and shapes subsequent biological outcomes [8]. A thorough grasp of these principles is expected to advance the effective application of NPs technologies across various domains, especially in medicine and drug delivery [6]. In the present review, the authors examine drug delivery platforms based on NMOFs employing diverse surface functionalization strategies designed to circumvent these biological hurdles.

## MOF modification strategies

MOF modification strategies are categorized as in situ functionalization and PSM. In situ functionalization incorporates functional groups or modulators during MOF self-assembly, directing crystal growth and enhancing crystallinity, though it may be limited by functional group incompatibility with solvothermal conditions and difficulty in introducing multiple functionalities [50, 54]. PSM involves covalent, coordinative, or non-covalent chemical transformation of pre-synthesized MOFs under mild conditions, preserving topology and porosity while enabling introduction of sensitive groups [55]. PSM is further classified into four types: covalent (e.g., amide bond formation, click chemistry), coordinative [56] (metal complex binding to open sites), non-covalent [57] (electrostatic interactions, hydrogen bonding, π-π stacking, van der Waals forces), and multilayer/hybrid PSM [58]. PSM efficiency depends on reactive site accessibility, governed by pore aperture, diffusion kinetics, solvent polarity, and competing species.

## PSM of MOFs

**Scheme 2:**
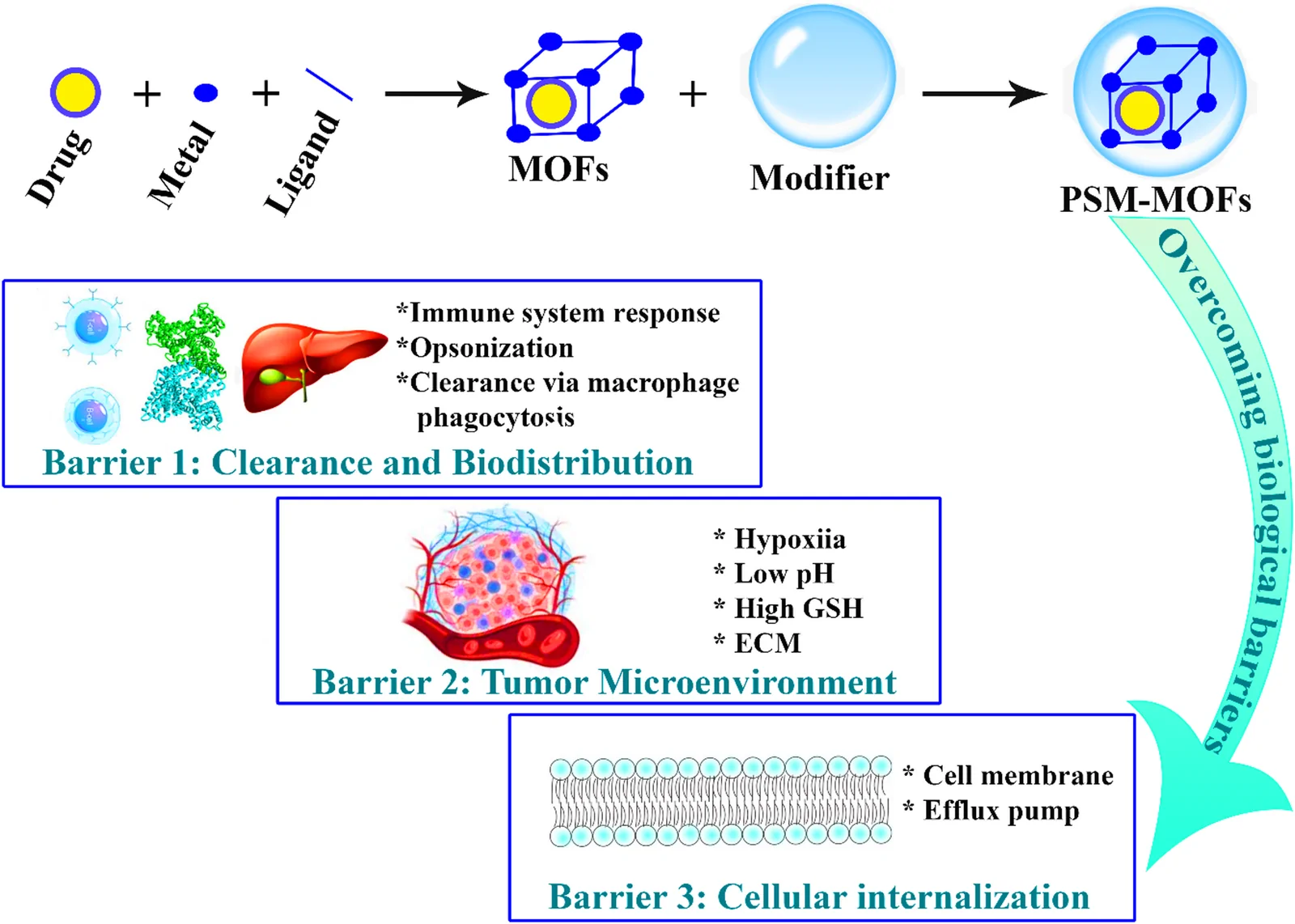
PSMs of MOFs for overcoming biological barriers

PSMs, including functional coatings, modulate the stability and blood circulation kinetics of NMOFs, prevent the release of toxic constituents during in vivo application, and enable targeted delivery. Beyond size and colloidal stability, biophysical attributes such as surface hydrophilicity, ligand type, and ligand density govern NMOF interactions with proteins, lipids, ions, and other physiological components. Surface modifications can alter particle morphology, charge, and size, thereby influencing cellular internalization mechanisms [59]. Consequently, PSMs overcome the surface limitations of NMOFs in biological environments **(**Scheme 2**).** Furthermore, whereas drug release from MOFs is typically regulated by drug–matrix interactions, PSMs provide a more effective strategy for achieving sustained and decelerated release kinetics [60].

**Scheme 3:**
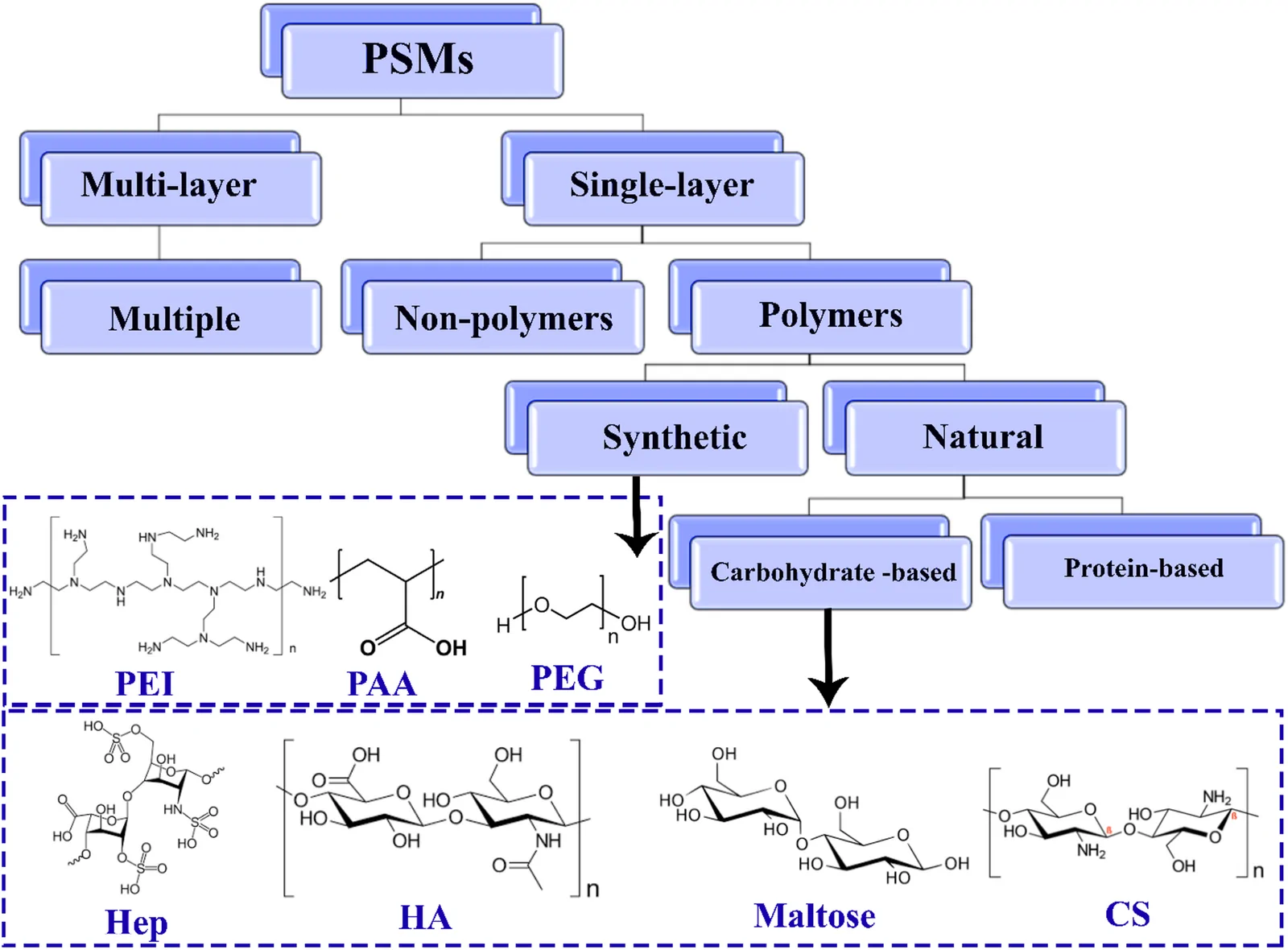
The classification of compounds for MOF surface modification

The classification of compounds for MOF surface modification encompasses single-layer and multi-layer architectures. Single-layer configurations are divided into polymeric and non-polymeric modifiers. Polymeric modifiers consist of long, flexible, entangled chains enabling viscoelasticity, stimulus-responsive swelling or shrinkage, and film-forming capabilities [61], which are unattainable with non-polymeric solid particles [62]. Polymeric single-layer modifiers further subdivide into natural and synthetic polymers [63], while non-polymeric materials form a distinct category. Natural polymers exhibit biocompatibility, biodegradability, and bioactivity but are limited by batch variability, potential immunogenicity, and low mechanical strength [64]. Synthetic polymers offer high reproducibility, tunable properties, and immune evasion, though some are non-biodegradable, bioaccumulative, and may require chemical modification to reduce cytotoxicity [63] **(**Scheme 3**).**

Each PSM strategy involves distinct trade-offs, and no single modifier is universally ideal. Peptides enable specific targeting but may undergo rapid degradation. Polyethylene glycol (PEG) prolongs circulation time yet can elicit anti‑PEG immune responses. Hyaluronic acid (HA) facilitates tumor targeting but suffers from variability in molecular weight and purity. Polydopamine (PDA) coatings are versatile but prone to batch‑to‑batch variability. Lipid coatings and cell membrane camouflaging provide excellent biocompatible stealth; however, maintaining membrane integrity while preventing drug leakage remains a significant challenge. Rational design therefore requires matching the selected PSM strategy to the intended therapeutic objective while addressing its inherent limitations. Table 1 summarizes the key merits and drawbacks of each modifier. In the following, we introduce several models categorized into four distinct modification types, each outlining specific strategies to overcome biological barriers and achieve sustained drug release.

**Table 1:** Merits and drawbacks of PSMs of NMOFs

Type of PSMs				Ex.	Merits	Drawbacks
**Single-layer modifiers**	**Polymers**	**Natural polymers**	**Carbohydrate**	Hep	* Improve colloidal stability * Reduce opsonization * Reduce systemic toxicity * Target CD44 receptors and antithrombin III * Improve cellular uptake	* Anticoagulant activity and requiring careful dose control * Batch-to-batch variability and reduce reproducibility * Potential immunogenicity * High cost
				HA	* Improve colloidal stability * Increase surface hydrophilicity * Prolonge blood circulation * CD44‑mediated active targeting	* Enzymatic degradation, * High molecular weight may hinder diffusion * Batch-to-batch variability
				Maltose	* Increase surface hydrophilicity * Reduce opsonization * Low cytotoxicity * Improve colloidal stability	* Covalent attachment of maltose require activation of the MOF surface * Require fusion protein for targeting * Stability concerns
				CS	* Biodegradable and pH responsive gatekeeping behavior * Positive zeta potential and promote cellular uptake	*Insoluble and stability concern at pH = 7.4→limiting itravenous use * Batch-to-batch variability
			**Protein**	AFT	*Transferrin receptor 1‑mediated active targeting *Improved biocompatible	*Poor Drug Retention and Leakage *Potential immunogenicity *Batch-to-Batch Variability
				BSA	*GP60 receptor‑mediated transcytosis *Stabilizes the NPs * Reduces opsonization, * Prolongs circulation time *Low-cost	*Stability and Denaturation Risks *Potential for Opsonization and Rapid Clearance *Batch-to-Batch Variability
				Gelatin	* Hydrophilicity and steric stabilization * Long-acting biodegradable * Low cost	* Potential immunogenicity, * Batch-to-batch variability * Residual glutaraldehyde crosslinker toxicity
		**Synthetic polymers**		PEG	* Increase steric stabilization *Increase surface hydrophilicity * Reduce opsonization * Avoid rapid immune clearance * Passive target via the EPR effect	*Induce anti-PEG antibodies and immune responses * Chain length dependency for long-term stability * Reduced cellular uptake at early time points
				PEI	*Efficient endosomal escape	* Intrinsic cytotoxicity * Need for charge neutralization
				PAA	*Improved biocompatible * Enable pH-triggered release	*Poor Mechanical Properties * Low Biodegradability * Pulmonary and Inhalation Toxicity
	**Non-polymers**			Lipid	* Improve biocompatibility * Improve cellular interactions * Sustained drug release behavior	*The oxidation of liposomes by singlet oxygen interferes with the outcomes of PDT.
				Biomimic	*Colloidal stability *Premature cargo release *Excellent immune evasion *Reduced systemic toxicity * Sustained drug release behavior	* Incomplete or uneven coating * Potential immunogenicity * Strict storage conditions * Scalability challenge * Batch-to-batch variability
				SiO_2_	*Controllable porosity * Improve colloidal stability * Low cytotoxicity * High surface area facilitate high drug loading	* Complex and multi-step synthesis *Non linear and unpredictable shell formation * Higher drug leakage during further functionalization
				Carbon	*Colloidal stability * Minimizes opsonization *Prolonging systemic circulation	*Poor biodegradability * Batch-to-batch variability * Risk of blockage of MOF pores
**Multi-layer modifiers**	**Multiple**				In general, * Enhanced multifunctionality *Superior Drug Release Control *Improved Colloidal Stability and Biocompatibility* *Synergistic Therapeutic Effects* *Broader Biological Barriers Penetration*	*Increased Synthetic Complexity *Potential for Interfacial Incompatibility *Characterization Challenges *Diminished Payload loading capacity *Higher Cost and Scalability Issues *Unpredictable In Vivo Fate

## Natural polymers

Natural polymers are biologically derived macromolecules stabilized by hydrogen bonding, selected for bioactivity and safety. They are classified into carbohydrate‑based (e.g., HA, heparin (Hep), chitosan (CS)) and protein‑based materials (e.g., albumin, apoferritin). Surface polar groups (‒OH, ‒NH₂/‒NHCOCH₃, ‒COOH, ‒OSO₃^−^) confer high hydrophilicity, pH‑responsive charge, and capacity for electrostatic complexation and biomolecular recognition, enabling receptor‑mediated targeting, biodegradability, cell‑specific recognition, and mucoadhesion [65]. An overview of these categories in MOF‑based systems and their tumor therapy applications is provided in **Supplementary Table S1**.

### Carbohydrate-based materials

Carbohydrate-based materials (HA, CS, Hep) are linear, hydrogen-bond-stabilized polysaccharides with high hydrophilicity [66, 67]; CS provides pH-responsive cationic character (pKa:~6.3‒6.5), HA is polyanionic, and Hep exhibits high negative zeta potential from dense sulfation. These functional groups enable covalent crosslinking, electrostatic complexation, and biomolecular recognition, governing colloidal stability, mucoadhesion, and hydrogel/nanocoating formation, while surface properties are linked to monosaccharide composition, glycosidic linkage pattern, sulfation degree, and derivatization [66].

Hep, a highly sulfated linear glycosaminoglycan, possesses dense O-sulfate, N-sulfate, and carboxyl groups that confer strong polyelectrolyte character and high negative zeta potential [68]. These anionic moieties enable electrostatic interactions with antithrombin III and CD44 receptor binding, supporting anticoagulation and active cell targeting [69]. Its conformational flexibility and sulfation pattern govern anticoagulant activity, CD44-mediated recognition, biocompatibility, and drug delivery utility [70].

**Scheme 4:**
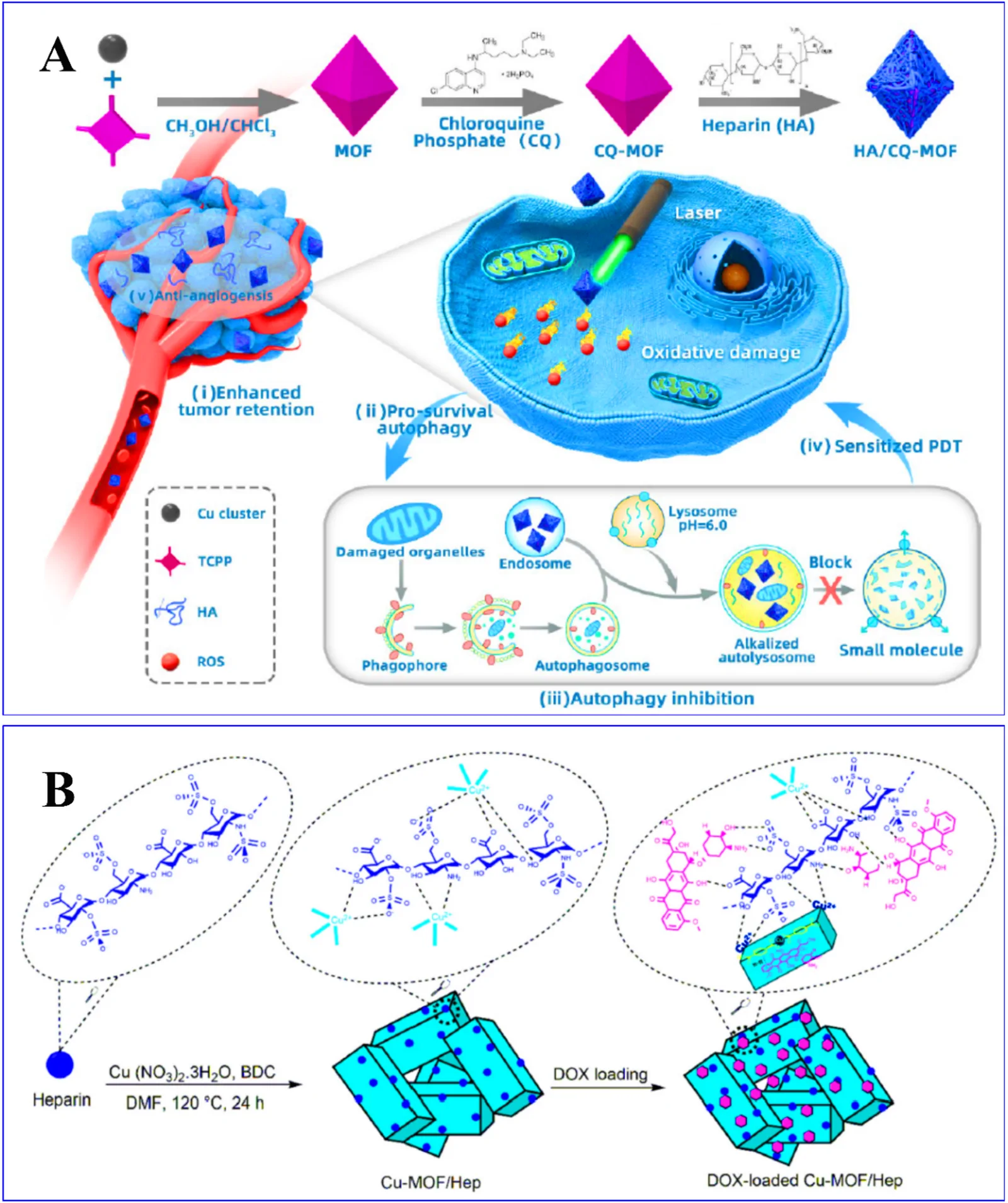
**(A) i.** Hep coating confers NPs with prolonged blood circulation time and subsequently enhanced tumor retention. **ii.** Hep/CQ-MOFs internalized by cancer cells produce abundant cytotoxic ROS under laser irradiation, which leads to oxidative damage and activation of prosurvival autophagy in cancer cells. **iii**,** iv.** CQ encapsulated in Hep/CQ-MOF alkalized the formed autolysosome and thus blocked the postautophagy pathway to enhance PDT. **V.** Antiangiogenesis effect of Hep/CQ-MOF. from ref [71]. with permission **(B)** Illustration of the synthesis of Cu-MOF/Hep nanomedicine, from ref [70]. with permission

In Sun et al., a chloroquine-loaded photosensitive porphyrinic NMOF was functionalized with Hepvia coordination bonding between copper clusters and Hep’s sulfate, carboxyl, and hydroxyl groups **(**Scheme 4A**).** Hep coating increased hydrophilicity, shifted zeta potential from positive to negative, enhanced colloidal stability, reduced hepatic clearance, and improved cellular uptake in 4T1 cells. The formulation enabled lysosomal accumulation, chloroquine-mediated autolysosomal alkalization, and elevated intracellular ROS upon laser irradiation. Consequently, it exhibited prolonged circulation, enhanced tumor accumulation via the EPR effect, antiangiogenic activity (reduced CD31), and superior tumor growth inhibition with increased apoptosis and autophagy inhibition (LC3 II and p62 accumulation). Hep functionalization thus optimizes pharmacokinetics, tumor homing, cellular uptake, ROS generation, chloroquine-mediated blockade of protective autophagy, and direct antiangiogenesis in photodynamic therapy (PDT) [71].

In a related study, coordination bonding between unsaturated copper sites on a Cu-MOF and heparin’s functional groups enabled stable covalent attachment, confirmed by FT‑IR and XRD **(**Scheme 4B**).** The Hep‑modified nanocarrier exhibited a positive zeta potential (+ 29.3 mV), ~ 100 nm hydrodynamic diameter, narrow size distribution, and ≥ 72 h colloidal stability in water. The hydrophilic Hep shell enhanced doxorubicin (DOX) loading (65.5% vs. 60.2% for unmodified MOF) and imparted pH‑responsive release (> 90% at pH 5, 41 °C within 96 h; <20% at pH 7.4, 37 °C). Positive surface charge enabled electrostatic attraction to cancer cell membranes, while Hep facilitated CD44 receptor recognition on MCF‑7 cells. Consequently, the DOX‑loaded heparin‑MOF conjugate showed superior cytotoxicity (IC₅₀: ~10 µg/mL vs. ~20 µg/mL for uncoated) and higher apoptosis (63% vs. 42% for unmodified MOF and 18% for free DOX). Nanoscale dimensions (~ 100 nm) enabled passive tumor accumulation via the EPR effect, while Hep coating minimized nonspecific interactions and potentially counteracted multidrug resistance. Overall, Hep functionalization improved physicochemical stability, conferred pH‑sensitive drug release, enabled CD44‑directed active targeting, and enhanced apoptotic efficacy [70].

HA is a linear, non-sulfated glycosaminoglycan of repeating D-glucuronic acid and N-acetyl-D-glucosamine disaccharides. Its carboxyl, N-acetyl, and hydroxyl groups confer hydrophilicity, viscoelasticity, and polyanionic character via hydrogen bonding and electrostatic interactions [72]. As a biocompatible, biodegradable extracellular matrix polysaccharide, HA is commonly used to modify MOFs [73, 74], providing prolonged circulation, stable coordination bonding, enhanced colloidal stability, CD44‑mediated tumor targeting, and hyaluronidase‑triggered intracellular release [69, 75]. A limitation is variable CD44 expression, reducing therapeutic response in tumors with low CD44 levels.

**Scheme 5:**
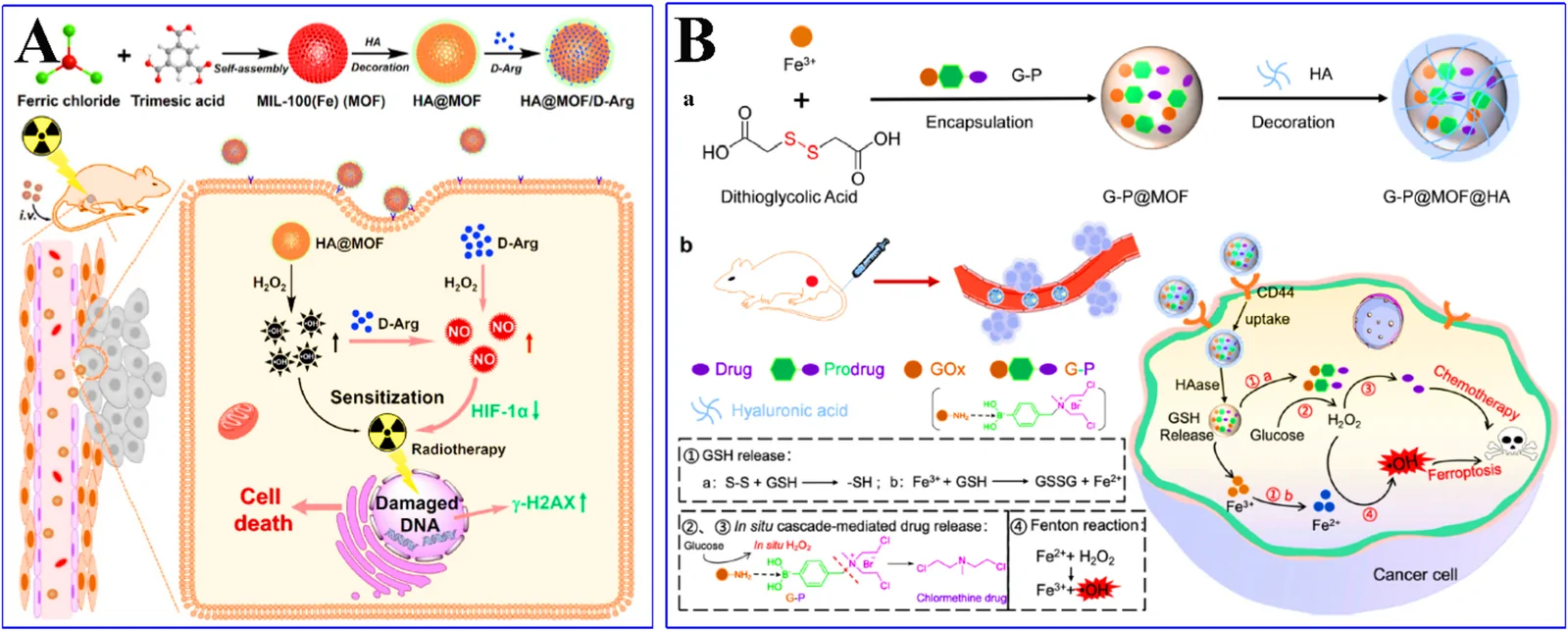
**(A)** Schematic illustration of the synthetic route to HA@MOF/D-Arg used for sensitizing osteosarcoma to radiotherapy, from ref [76]. with permission, **(B)** Schematic illustration of the preparation of G‒P@MOF@HA and the cascade reactions for cancer therapy, from ref [77]. with permission

Accordingly, MIL‑100(Fe) functionalized with HA via coordination bonding yielded uniform NPs with high surface area and sustained colloidal stability **(**Scheme 5A**)**. The HA shell enabled active targeting of CD44‑expressing osteosarcoma cells. Ferric ions mediated Fenton reactions with endogenous H₂O₂ to generate ROS. Upon loading with D‑arginine, the nanoplatform produced nitric oxide and, under low‑dose X‑ray irradiation, generated enhanced ROS and nitrogen species. Under hypoxia, this combination down‑regulated hypoxia‑inducible factor‑1α, induced DNA damage (γ‑H2AX), and promoted apoptosis, overcoming radioresistance. In a murine osteosarcoma model, intravenous administration of the D‑arginine‑loaded, HA‑decorated MOF with two low‑dose X‑ray exposures suppressed tumor growth, reduced tumor mass, extended survival to 60 days with negligible systemic toxicity, and overcame radioresistance and metastasis [76].

In another study, an iron-based MOF functionalized with HA via electrostatic adsorption produced NPs (~ 124.5 nm diameter) with low polydispersity, enhanced colloidal stability, and increased hydrophilicity **(**Scheme 5B**)**. The HA shell enabled selective CD44-targeted uptake. Upon internalization, elevated GSH triggered framework disassembly and release of glucose oxidase (GOx) and a chlormethine prodrug. GOx-generated H₂O₂ and ferrous ions from degraded MOF drove Fenton reactions to produce hydroxyl radicals, inducing ferroptosis via lipid peroxidation, while H₂O₂ activated the prodrug in situ. This dual mechanism yielded superior cytotoxicity, increased apoptosis, down-regulated GPX4, and suppressed tumor growth in a murine xenograft model without significant systemic toxicity. HA decoration thus conferred colloidal stability, active tumor targeting, and a cascade catalytic platform overcoming intratumoral H₂O₂ deficiency [77].

Maltose is a reducing disaccharide of two glucose units linked by an α (1→4) glycosidic bond, with eight hydroxyl donors conferring high polarity and extensive hydrogen-bonding capacity. Its free anomeric carbon imparts reducing sugar reactivity, enabling function as a surface-active polyol in polar interactions [78].

**Scheme 6:**
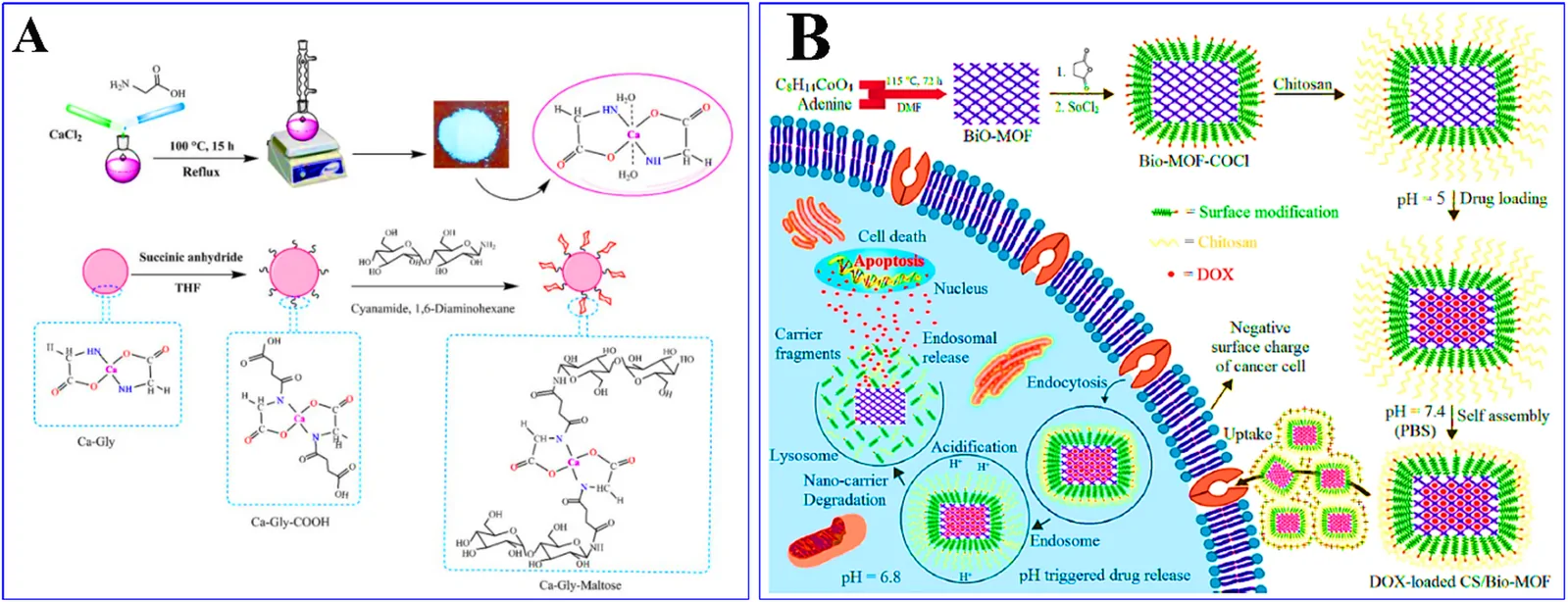
**(A)** Schematic illustration of the Synthesis pathway of Ca-Gly and surface modification and fabrication of Ca-Gly-Maltose, from ref [37]. with permission, **(B)** Schematic diagram of the DOX@CS/Bio-MOF formation and its drug release behavior at pH 6.8, from ref [34]. with permission

In this context, a Ca Gly-MOF was covalently modified with maltose via carboxylation and amide coupling, yielding increased hydrodynamic diameter, a shift from positive to slightly negative surface charge, reduced surface area, and retained spherical mesoporous morphology **(**Scheme 6A**)**. The maltose layer enabled high-affinity immobilization of an anti-VEGFR1 single-chain variable fragment fused to maltose-binding protein, conferring active tumor targeting. The nanoplatform co-loaded with prodigiosin and simvastatin exhibited pH-dependent release and synergistic cytotoxicity against VEGFR1-overexpressing cancer cells (IC₅₀ = 1.80 µg/mL; combination index < 1) and inhibited cell migration by > 80% relative to free drugs. Maltose functionalization thus enabled targeted, pH-responsive co-delivery, synergistically enhancing cytotoxicity and suppressing metastasis while overcoming key chemotherapy limitations [37].

CS is a linear cationic polysaccharide with primary amino (‒NH₂) and hydroxyl (‒OH) groups that confer pH‑responsive protonation (pKa :~6.3‒6.5) and electrostatic/hydrogen‑bonding capacity [79]. Its degree of deacetylation and semi‑crystalline structure determine surface functional group density, dictating reactivity, hydrophilicity, and suitability for chemical modifications in biomedical applications [80].

A cobalt‑adeninate‑butyrate Bio-MOF was covalently conjugated with CS via carboxylation, acyl chloride formation, and immobilization **(**Scheme 6B**).** The composite retained crystalline structure, exhibited reduced surface area, pH‑dependent surface charge (positive at acidic pH, negative at alkaline pH), and a homogeneous CS layer. It showed enhanced DOX loading and a strongly pH‑dependent release profile, with rapid drug liberation under mildly acidic conditions. The DOX‑loaded CS‑modified framework demonstrated potent cytotoxicity against breast cancer cells, outperforming free DOX, while the empty carrier was non‑toxic. The formulation induced higher apoptosis, enabled intracellular nuclear delivery, and exhibited minimal cobalt leaching. Thus, the CS‑capped bio‑MOF functioned as a biocompatible, pH‑responsive nanocarrier that significantly improved DOX delivery and anticancer efficacy [34].

### Protein-based materials

Apoferritin, albumin, and gelatin represent distinct natural protein systems with characteristic surface chemistries and structural organizations. Apoferritin (24-subunit hollow nanocage, 12 nm [81]), albumin (globular monomer, ~ 66 kDa, with free thiol Cys‑34 and 17 disulfide bonds [82]), and gelatin (denatured collagen lacking tertiary structure [83]) exhibit distinct surface chemistries. Apoferritin presents lysine ε-amines, carboxylates, and tyrosine phenols enabling bioconjugation. Albumin features charged and hydrophobic patches with amines, carboxyls, and thiols conferring ligand-binding and pH-dependent flexibility [84]. Gelatin displays randomly distributed arginine, lysine, and hydroxyproline residues bearing amines, carboxyls, and hydroxyls, imparting amphoteric, thermoreversible, and adhesive properties [85]. These surface functional groups—amines, carboxyls, thiols, and hydroxyls—determine each protein’s reactivity, charge profile, and solvent/ion/biomolecule interactions. These protein coatings furnish additional functional groups for conjugation, improve biocompatibility, protect MOFs from premature physiological degradation, and promote receptor-mediated cellular uptake, thereby enabling targeted therapeutic delivery [86].

(ZIF-8) was surface‑decorated with apoferritin via a water‑induced defect mechanism involving Zn‒N bond cleavage and coordination with protein N‑termini, yielding a spiky surface morphology with reduced area, high protein loading, and pH‑dependent release. This decoration enabled active targeting of transferrin receptor 1 on triple‑negative breast cancer cells, enhancing cellular uptake, cytotoxicity, and apoptosis while sparing normal cells. In a murine xenograft model, the nanovehicle accumulated at tumors, achieved high growth inhibition, and exhibited negligible systemic toxicity. Thus, apoferritin‑based spiky surface modification confers transferrin receptor 1‑mediated active targeting, hierarchical pH‑responsive release, and lysosome‑specific delivery, synergistically improving anticancer efficacy and minimizing off‑target effects [14].

**Scheme 7:**
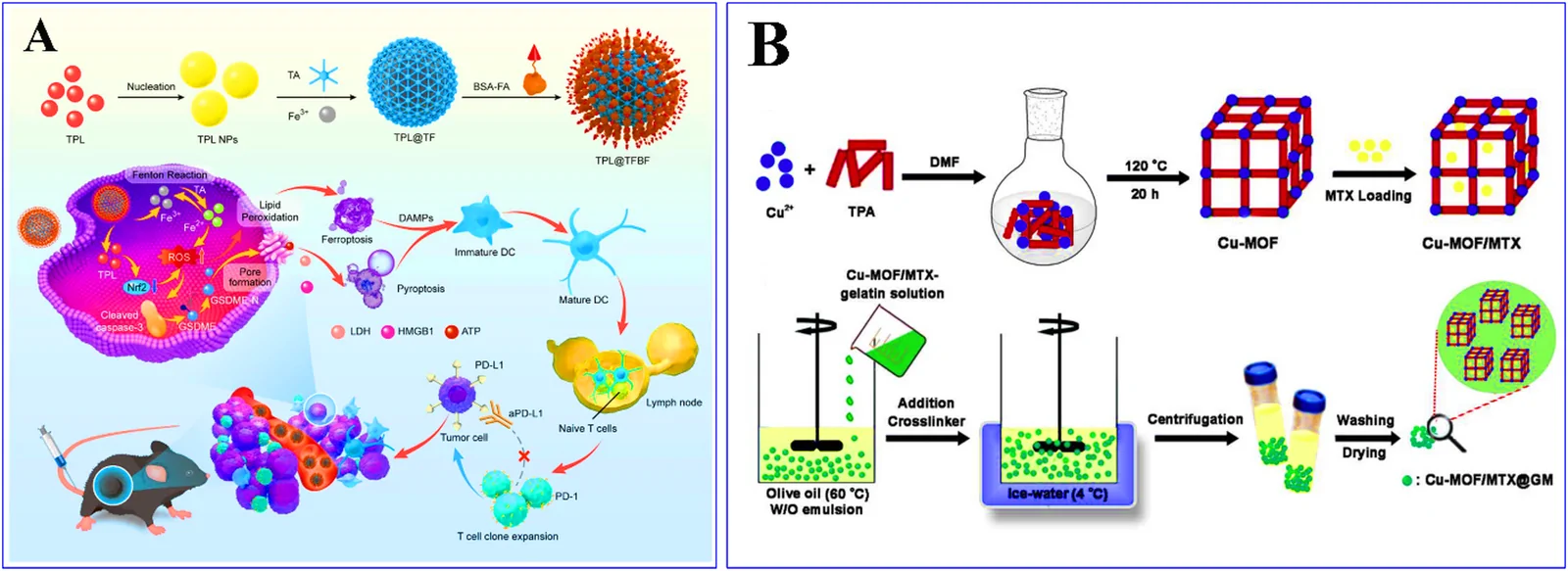
**(A)** Synthesis of TPL@TFBF and its cancer immunotherapy mechanism of inducing ferroptosis and pyroptosis, from ref [90]. with permission, **(B)** General procedure employed for encapsulating Cu-MOF/MTX with gelatin microspheres (Cu-MOF/MTX@GM), from ref [60]. with permission

Bovine serum albumin (BSA) is a cost‑effective, highly stable protein (583 amino acids) bearing carboxylic acid, thiol, and amino groups, approximately 21 metal‑binding sites, and close resemblance to human serum albumin [87]. BSA facilitates GP60 receptor‑triggered transcytosis, stabilizes NPs, prevents aggregation, reduces opsonization, and prolongs circulation time, enabling a dual‑receptor strategy that surpasses passive EPR [88, 89]. An iron‑tannic acid MOF coated with folic acid (FA)‑modified BSA via non‑covalent interactions yielded NPs with high triptolide loading, colloidal stability, and active targeting **(**Scheme 7A**)**. The BSA coating enables GP60‑mediated transcytosis, reduced opsonization, and prolonged circulation, complementing folate receptor‑mediated uptake. In the acidic TME, tannic acid reduces Fe^3+^ to Fe^2+^, driving the Fenton reaction to generate hydroxyl radicals, while triptolide inhibits Nrf2, suppresses GPX4, and depletes GSH, amplifying oxidative stress. This dual mechanism induces concurrent ferroptosis and pyroptosis, evidenced by caspase‑3 activation, gasdermin E cleavage, LDH release, and IL‑1β secretion, leading to dendritic cell maturation, CD4⁺/CD8⁺ T cell infiltration, and systemic antitumor immunity. In a murine melanoma model, the formulation achieved 65.6% tumor inhibition, near‑complete suppression of lung metastasis, 83% survival at 14 days, and negligible toxicity, demonstrating that the BSA‒FA coating enables targeted synergistic ferroptosis/pyroptosis to overcome low immunogenicity, drug resistance, and metastasis in melanoma immunotherapy [90].

Gelatin, a polydisperse polypeptide from collagen hydrolysis, features surface amines, carboxyl, hydroxyl, and minor sulfhydryl groups [91]. Gelatin microspheres (GMs) prepared via water‑in‑oil emulsification [92] and glutaraldehyde crosslinking [93] were used to encapsulate a methotrexate (MTX)‑loaded Cu-MOF **(**Scheme 7B**)**. The glutaraldehyde covalently bridges gelatin amino groups, forming a stable, pH‑responsive hydrophilic shell that preserved the composite’s spherical morphology and crystalline structureThe resulting pH‑responsive shell reduced burst release and yielded a tumor‑mimetic (pH 5.3, 41 °C) cumulative release of about 70% over 150 h versus < 35% at physiological pH 7.4/37°C. The coating enabled MTX to target folate receptors on MCF‑7 cells, enhancing cytotoxicity beyond free drug while the empty carrier remained nontoxic. Thus, gelatin microspheres confer colloidal stability, pH‑dependent release, and sustained, receptor‑mediated targeting, improving anticancer efficacy and reducing off‑target toxicity [60].

### Synthetic polymers

Synthetic polymers, valued for controlled and tunable performance, enable PEGylation of MOFs via covalent or coordinative bonding to surface metal sites or linkers. This imparts a dense, neutral, hydrophilic corona that enhances colloidal stability, suppresses protein adsorption and opsonization, and confers stealth behavior [94]. Consequently, systemic residence is prolonged, clearance by the MPS is evaded, passive targeting via the EPR effect is amplified, and non‑disruptive passage across endothelial and mucosal barriers is permitted, ultimately facilitating passive targeted therapeutic delivery [95].

Liang et al. described Fe‑Cu‑based MOFs bearing disulfide bridges and surface‑PEGylated with C18PMH‑mPEG, improving dispersibility, stability, and tumor accumulation via the EPR effect. In GSH‑overexpressing TMEs, disulfide cleavage depleted GSH (inactivating GPX4), released Fe^2+^/Cu^2+^ (catalyzing Fenton/Fenton‑like ^•^OH generation), and triggered DOX release (> 85%). Concomitant photothermal effects (808 nm) accelerated radical production and membrane permeabilization, inducing lipid peroxidation and ferroptosis, thereby achieving selective cytotoxicity against 4T1 cells, significant in vivo tumor inhibition, and minimal systemic toxicity via synergistic chemodynamic, photothermal, and chemotherapy [96].

**Scheme 8:**
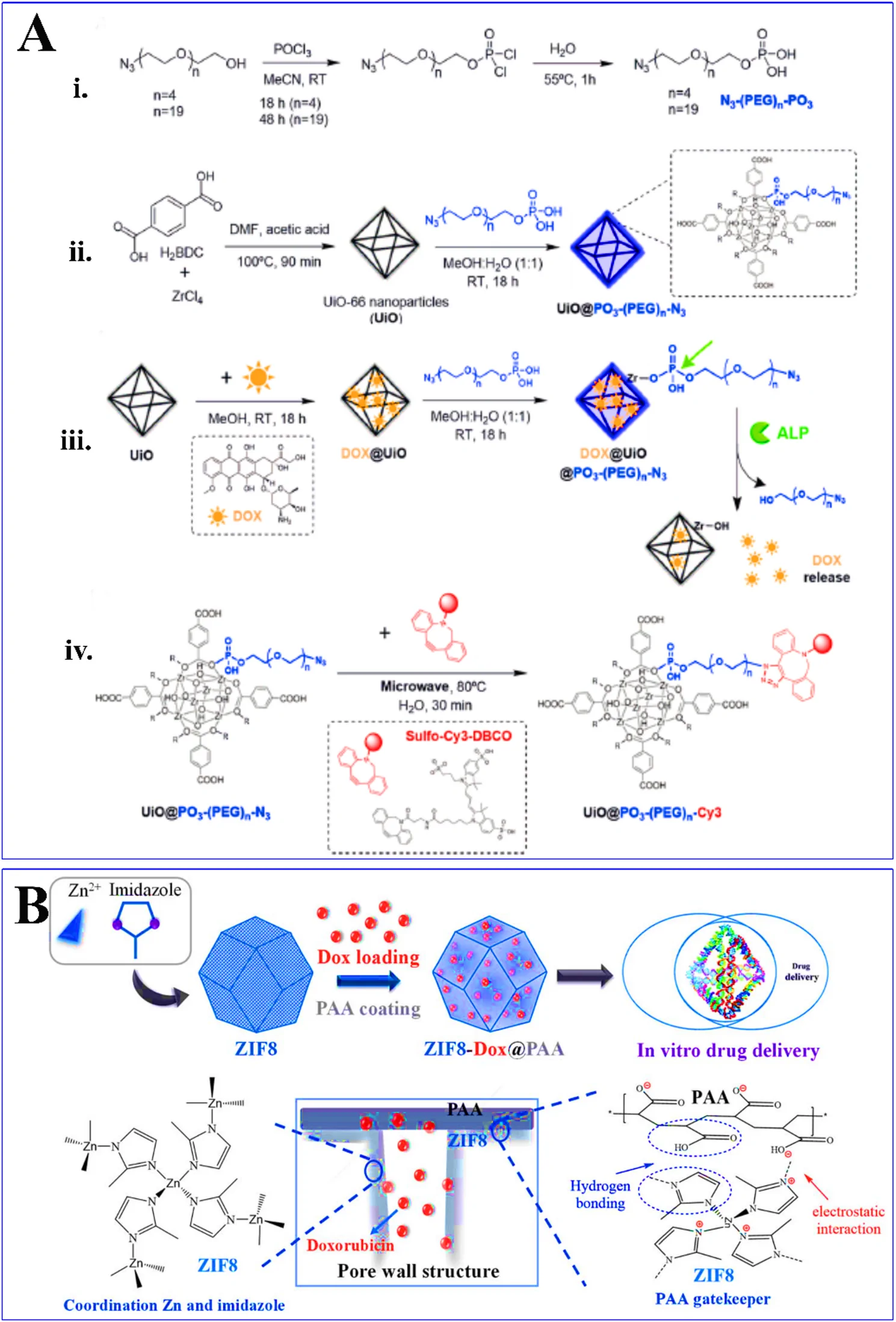
**(A)** (i) Simplified scheme of the synthetic route to N3-(PEG)*n*-PO3 ligands; (ii) Schematic illustration of the solvothermal synthesis of UiO-66 NMOFs (UiO) and the following PSM strategy to coat the UiO with N3-(PEG)*n*-PO3 ligands; (iii) Representation of the loading of UiO NPs with DOX, followed by their surface functionalization with the ligands, and the proposed DOX release triggered by the enzyme alkaline phosphatase (ALP); (iv) Scheme of the attachment of a fluorescent probe (Sulfo-Cy3-DBCO) to the surface of the UiO@PO3-PEG-N3 particles by using a copper-free microwave-assisted “Click” reaction, From ref [97]. with permission, **(B)** Diagrammatic representation of ZIF8-DOX@PAA’s pore wall structure and preparation as a vehicle for DOX encapsulation and pH-triggered release, From ref [98]. with permission

In other study, nanoscale UiO‑66 functionalized with N₃‑(PEG)₂₀‒PO₃ ligands via stable Zr‒O‒P coordination showed increased negative zeta‑potential, uniform size (~ 224 nm), and exceptional colloidal stability in PBS (> 2 weeks) versus unmodified UiO‑66 (Scheme 8A**)**. The PEG layer preserved crystallinity and porosity, while terminal azide groups enabled 98% efficient copper‑free click conjugation. The phosphate ester prevented premature DOX release (< 10% over 72 h without alkaline phosphatase). Enzymatic cleavage by alkaline phosphatase in HeLa cells (high expression) triggered markedly higher drug release and a four‑fold lower half‑maximal inhibitory concentration versus ALP‑low MCF7 cells. Thus, the PEG‑phosphate coating confers colloidal stability, prevents non‑specific leakage, enables enzyme‑selective release in ALP‑overexpressing cancer cells, and provides a clickable handle for further functionalization [97].

In other study, polyacrylic acid (PAA) functionalization of zeolitic imidazolate framework‑8 (ZIF-8) via electrostatic interactions and hydrogen bonding conferred aqueous dispersity, high DOX loading (62 mg/g), and intrinsic fluorescence (Scheme 8B**)**. The PAA shell acted as a pH‑responsive gatekeeper: at pH 7.4, strong electrostatic attraction formed a dense barrier limiting release to 5.7% at 30 h and 24.7% at 100 h; at pH 4.0, PAA protonation triggered polymer detachment and MOF degradation via Zn‒imidazole cleavage, accelerating cumulative release to 84.7% at 30 h. This prevented non‑specific leakage under neutral conditions while enabling selective, pH‑triggered release in acidic tumor compartments. Degradation byproducts (Zn^2+^, imidazolates) showed no significant cytotoxicity up to 50 µg mL⁻¹, confirming biocompatibility. Thus, the PAA‑decorated ZIF8 platform integrates high drug loading, fluorescent tracking, and pH‑responsive release as a theranostic strategy [98].

Polyethyleneimine (PEI) possesses high amine density, conferring positive surface charge, hydrophilicity, biocompatibility, and a proton sponge effect under acidic conditions. Low-pH protonation facilitates endocytosis and endosomal escape [99], while electrostatic interactions with negatively charged vasculature promote tumor penetration [100]; inherent toxicity of branched PEI can be mitigated by surface charge neutralization strategies. In a specific formulation, a nanoscale NH₂‑UiO‑66 core was sequentially coated with an alginate interlayer and a PEI shell covalently conjugated to glycyrrhetinic acid (GA) via amide bonds. The PEI‑GA shell imparted a less negative zeta‑potential (− 6.2 mV), high DOX loading (23 wt%, 97% efficiency), and pH‑dependent release (65‒76% over 72 h at pH 4‒5.4 vs. 30% at pH 7.4). GA mediated active targeting to glycyrrhetinic acid receptors on HepG2 cells, yielding a ten‑fold lower IC₅₀ (6.89 µg/mL). Upon endosomal acidification, PEI protonation triggered the proton sponge effect, causing endosomal rupture and cytosolic release, increasing apoptosis (27.5%) and sub‑G1 cells twenty‑fold. Empty carriers showed minimal cytotoxicity. Thus, the PEI‑GA shell imparts pH‑responsive release, active targeting, and efficient endosomal escape for hepatocellular carcinoma (HCC) therapy [101].

## Non-polymeric materials

Non‑polymeric modifiers (silica [102], phospholipid‑based [103], carbon‑based materials [104]) deposit on MOF surfaces as discrete, rigid entities (particles, sheets, vesicles) without long‑chain entanglement or viscoelastic behavior. Silica forms a mesoporous shell enhancing hydrothermal/chemical stability. Liposomes assemble into vesicular bilayers enabling cargo delivery via lipid phase behavior. Graphene oxide (GO) coats as thin two‑dimensional sheets through electrostatic interactions and hydrogen bonding, imparting conductivity, hydrophilicity, and defect sites [105].

### Phospholipid-based materials

Phospholipid bilayer coatings on MOFs confer high biocompatibility, attenuate toxicity, preserve structural stability, enable sustained drug release, enhance cellular internalization, and prolong circulation via immune evasion [106]. A complementary biomimetic approach using cell membrane cloaking (erythrocyte, macrophage, stem cell, or cancer cell membranes) functionalizes NMOFs to extend systemic residence, facilitate membrane-mediated interactions, improve tumor-cell targeting, reduce off-target drug accumulation, and augment endocytosis [107].

#### Liposomes

**Scheme 9:**
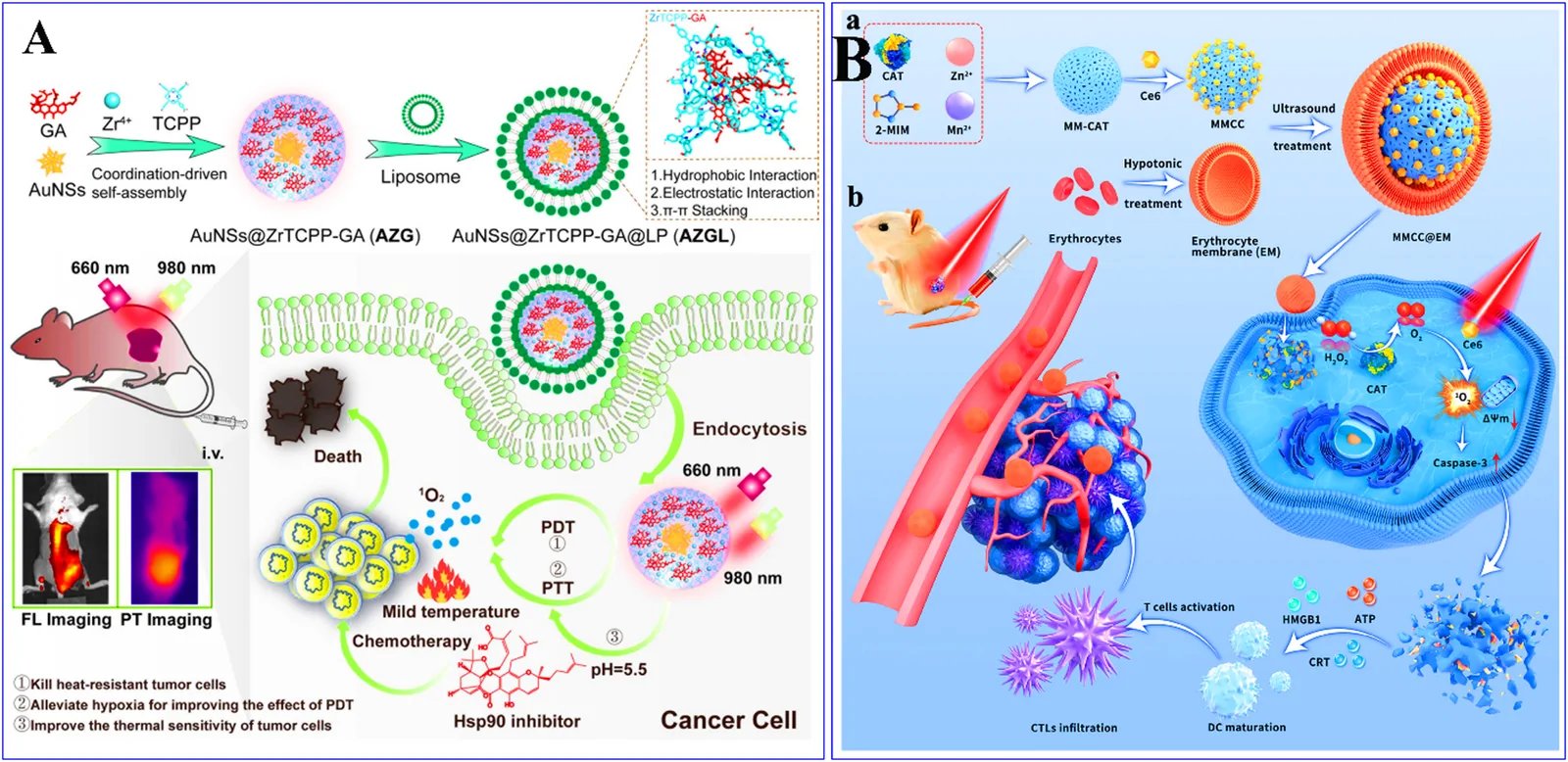
**(A)** The synthetic illustration of AZGL nanocomposite formation and its synergistic Photothermal therapy (PTT)-PDT-chemotherapy for breast cancer. From ref [109]. with permission. **(B)** Schematic illustration of MMCC@EM nanotheranostics for FL/PA duplex imaging- escorted self-oxygenation PDT. (a) The synthetic process of MMCC@EM. (b) The therapeutic mechanism mediated by MMCC@EM. From ref. From ref [116]. with permission

Liposome‑coated MOFs integrate a fluid phospholipid bilayer with a rigid crystalline MOF via phosphate‒metal coordination, electrostatic forces, or covalent linkers [103]. The densely functionalized MOF exterior enables high‑capacity conjugation of targeting ligands and steric polymers, surpassing liposomal loading limits and enhancing receptor‑mediated binding. These hybrids combine liposome biocompatibility with MOF porosity and chemical versatility, establishing nanocarriers with spatiotemporally controllable surface properties and release behavior [108]. Accordingly, the AuNS@ZrTCPP‑GA (AZG) nanocomposite was surface‑functionalized with a PEGylated liposome via non‑covalent electrostatic and hydrophobic interactions, shifting zeta potential from − 35.9 mV to − 16.9 mV (Scheme 9A**)**. The coating increased hydrodynamic diameter, ensured colloidal stability over seven days, preserved photothermal conversion efficiency (~ 27.5%) and singlet oxygen generation (98.47% ADPA absorption decrease), and conferred pH‑dependent release of gambogic acid (GA) and TCPP (54.2%/47.3% at pH 5.5 vs. 22.2%/13.4% at pH 7.4 over 24 h) via acid‑induced MOF degradation. The PEGylated liposome shell reduced macrophage internalization, prolonged circulation, and enhanced tumor accumulation through the EPR effect, enabling synergistic photothermal‑photodynamic‑chemotherapy with mild hyperthermia (~ 9.1 °C temperature rise) and negligible systemic toxicity (minimal hemolysis, stable body weight and serum biochemistry, limited histopathological injury) [109].

In a similar study, lipid corona anchored onto MIL-100(Fe) NMOFs via rapid solvent‑exchange deposition (non‑covalent phosphate‒iron coordination, hydrophobic interactions) preserved crystallinity, porosity (~ 1500 m²/g BET), and size (~ 240 nm), shifted zeta potential to consistently negative (DOPC) or near‑neutral (PEGylated), enhanced colloidal stability, retarded framework degradation (trimesate release ~ 15.5% to ~ 10% at 1 h), and prolonged cargo release (Gem‑MP release 78% to 50% at 6 h in 3 mM PBS) by restricting phosphate diffusion. The corona conferred a stealth effect (albumin adsorption ~ 50 to ~ 5 µg/mg; macrophage uptake reduced up to 3.6‑fold), while drug‑loaded, lipid‑coated particles retained potent anticancer activity, internalized into SKOV3 cells, and surpassed free Gem‑MP cytotoxicity [106].

#### Biomimetic camouflages

MOFs functionalized with cell membrane coatings emulate intrinsic cellular characteristics to extend circulatory half‑life, enhance tumor accumulation, and reduce macrophage‑mediated clearance, with diverse membrane origins (cancer cells, erythrocytes, macrophages) critically assessed for biomimetic camouflage [110].

Cancer cell membrane‑coated MOFs enable homologous tumor targeting via retained surface antigens, minimizing off‑target effects [111]. To this end, a zirconium‑based porphyrinic MOF co‑encapsulating the TLR7 agonist R837, functionalized with triphenylphosphonium for mitochondria targeting, and cloaked with 4T1 cancer cell membranes via non-covalent interactions (zeta = − 19.23 mV, ~ 216 nm) preserved crystallinity and loading. Upon ultrasound irradiation, the platform generated ROS, inducing immunogenic cell death. Together with R837, it promoted dendritic cell maturation and pro‑inflammatory cytokines. Combined with anti‑CTLA‑4 blockade, treatment reduced regulatory T cells, increased intratumoral CD8⁺ T cells to 61.5%, suppressed primary/metastatic tumor growth, extended survival to 40% at 50 days, and established durable immunological memory, demonstrating homologous targeting and mitochondrial‑localized sonodynamic immunotherapy synergistic with immune checkpoint blockade [112].

In another report, Fe(SS) MOF (Fe^3+^ coordinated with dithiodiglycolic acid) encapsulating GOx and DOX was enveloped in 4T1 cancer cell membranes via non‑covalent hydrophobic/electrostatic forces, forming a core‒shell architecture (~ 13 nm shell). This biomimetic coating shifted zeta potential from − 18.7 mV to − 24.9 mV, increased hydrodynamic diameter to ~ 190 nm, maintained colloidal stability for two weeks, and preserved homologous recognition capabilities, conferring immune evasion and homotypic tumor targeting. In an orthotopic breast cancer model, the nanocarrier enhanced intracellular uptake and tumor localization (maximal fluorescence at 12 h). Within the TME, disulfide bridges depleted GSH, while GOx generated H₂O₂ to amplify Fenton reactions and ROS, inducing ferroptosis and glycolysis suppression, leading to immunogenic cell death, reprogramming of the lactate‑dominated immunosuppressive milieu, significant tumor growth suppression, and negligible systemic toxicity. Thus, cancer cell membrane coating circumvents non‑specific clearance, enables homologous targeting, and optimizes ferroptosis‑based combination chemotherapy [113].

Blood cell membranes confer stealth properties via unique proteins, glycans, and sialic acid residues, reducing RES clearance and prolonging circulation [114]. Red blood cells (RBCs) membrane coating enhances NPs stability but possesses limited intrinsic tumor-targeting capacity, though RBCs’ oxygen transport can alleviate tumor hypoxia [115]. To counteract tumor hypoxia—which compromises oxygen‑dependent PDT—a zeolitic imidazolate framework encapsulating chlorin e6 (photosensitizer) and catalase was camouflaged with erythrocyte membrane (EM) via non‑covalent interactions, preserving CD47, colloidal stability (~ 231 nm), and pH‑responsive release **(**Scheme 9B**).** The catalase generates oxygen from endogenous H₂O₂, alleviating hypoxia and enabling effective self‑oxygenation PDT upon laser irradiation, while the membrane coating enhances circulation and tumor accumulation via the EPR effect, achieving antitumor efficacy with minimal systemic toxicity [116].

Camouflaging synthetic MOF cores with macrophage membranes (MM)—exploiting their inflammation‑homing properties—produces biological‑synthetic hybrid systems capable of navigating complex physiological barriers with high precision. MM vesicles were used to coat piperine (PIP)-loaded MIL-100(Fe) NPs via non-covalent electrostatic attraction and amine-carboxyl conjugation. The resulting core‒shell architecture (outer lipid bilayer ~ 10 nm) exhibited increased hydrodynamic diameter (98 nm to 150 nm), a zeta potential of − 32 mV, and colloidal stability for ≥ 10 days. Drug release followed the Korsmeyer‒Peppas model, with pH-responsive elution (25% at pH 5.0 vs. 7.2% at pH 7.4 over 14 days). The MM coating conferred immune evasion and integrin-mediated active targeting, reducing IC₅₀ values 4- to 17-fold relative to free PIP in breast cancer cell lines [117].

In a separate investigation, mesenchymal stem cell membranes were coated onto Cu-MOF NPs via non-covalent electrostatic and hydrophobic interactions using sonication and extrusion, increasing hydrodynamic diameter (122 nm to 164 nm) and inverting zeta potential (+ 20.95 mV to − 27.45 mV). The biomimetic coating conferred colloidal stability, immune evasion, and prolonged circulation, enabling preferential tumor homing and a two-fold enhancement of intracellular uptake in melanoma cells, where accumulated cytotoxic copper ions induced apoptosis and overcame the tumor’s biological barrier, resulting in superior anti-tumor efficacy [118].

Extracellular vesicle membranes (EVMs) constitute another class of biomimetic membrane. These endogenous, lipid-based vesicles, present in various bodily fluids, are non-immunogenic and serve essential functions in intercellular communication [119]. EVMs derived from MDA-MB-231 tumor cells were self-assembled onto protein-loaded ZIF-8 NPs via ultrasonication and extrusion, driven by non-covalent interactions. The coating yielded a surface charge comparable to natural EVs, achieved high protein loading efficiency (~ 94%) and capacity (~ 41%), retained pH-responsive release, maintained colloidal stability for ≥ 3 days, and reduced serum protein opsonization by approximately 6‑fold relative to uncoated particles. EVM coating enabled immune evasion (macrophage uptake reduced to ~ 30% of uncoated controls), conferred strong homotypic targeting (2‒8‑fold higher uptake in source tumor cells), and enhanced intracellular protein transduction efficiency (~ 77.5%). In a mouse xenograft model, systemically administered gelonin-loaded NPs demonstrated sustained tumor accumulation (up to 72 h), 4‑fold higher tumor fluorescence than uncoated counterparts, substantial tumor growth inhibition, and minimal systemic toxicity [120].

### Carbon-based materials

The functionalization of MOFs with GO or reduced graphene oxide (rGO) exploits oxygen-containing groups (hydroxyl, epoxy, carboxyl) and π-conjugated networks to form covalent and non-covalent interactions [121, 122]. GO is hydrophilic and negatively charged with a mixed sp²/sp³ structure, whereas rGO exhibits fewer oxygen groups, restored sp² domains, enhanced conductivity, and greater hydrophobicity [123]. These interactions induce physicochemical changes in the composites, including altered morphology, size (tens to > 150 nm), and surface charge inversion (e.g., positive to negative zeta potential) [124], Such modifications enhance cellular uptake (via reduced size), reduce opsonization, and prolong circulation, while the low immunogenicity, high surface-area-to-volume ratio, and tunable chemistry of the carbon components govern biodistribution, uptake pathways, and clearance profiles [123].

**Scheme 10:**
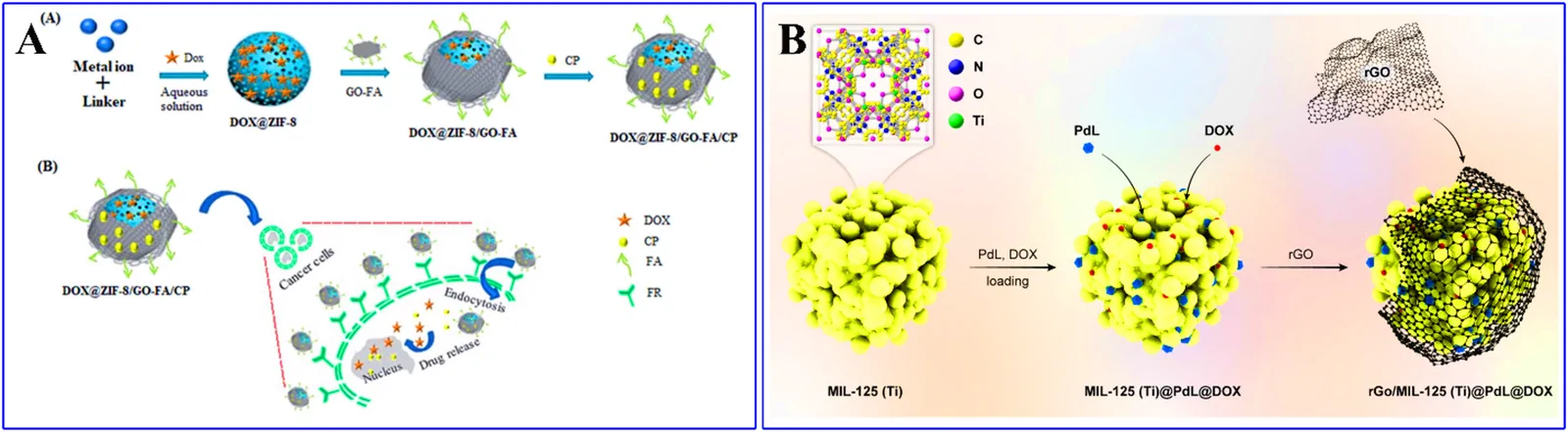
**A**: Protocol illustrations for the synthesis of DOX@ZIF-8/GO‒FA/CP. from ref [125]. with permission. **B.** A schematic illustration of MIL-125‑based nanocomposite fabrication. from ref [126]. with permission

In this context, Singh et al. engineered ZIF‑8 surfaces with FA‑functionalized GO (GO‑FA) via Zn^2+^ coordination, yielding negatively charged, crystalline spherical particles (45–60 nm). DOX (87% efficiency, pore‑loaded) and cyclophosphamide (53%, GO‑adsorbed via H‑bonding/π‒π interactions) exhibited pH‑dependent release (76‒80% at pH 5.6 vs. 50% at pH 7.2) due to acid‑labile ZIF‑8 (Scheme 10A**)**. The FA‑targeted nanocarrier enabled selective uptake and synergistic cytotoxicity in folate receptor‑positive breast cancer cells (combination indices 0.29‒0.75), with negligible toxicity to normal cells. This pH‑responsive, dual drug delivery system (DDS) effectively reduced off‑target effects and improved therapeutic efficacy [125].

Bagherzadeh et al. surface‑engineered MIL‑125(Ti) with reduced GO, a Pd (II) carboxamide complex, and DOX, stabilized by π‒π stacking, hydrogen bonding, and van der waals forces (Scheme 10B**)**. The nanocomposite (~ 156 nm, − 10 mV, 48.9% DOX loading) exhibited pH‑responsive, sustained release (~ 40% less drug at pH 7.4 over 120 h vs. free drug). The Pd complex reduced cytotoxicity in HEK‑293 cells by > 15%, maintained an AST: ALT ratio below the healthy threshold (1.46), and preserved liver morphology, indicating high biocompatibility. Additionally, antibacterial activity against S. aureus increased ~ 200% relative to DOX alone. Overall, this system mitigated burst release, off‑target toxicity, and poor liver biocompatibility through synergistic, pH‑sensitive nanocarrier design [126].

### Silica

Silica (SiO₂) comprises a three-dimensional network of tetrahedral silicon atoms bridged by oxygen atoms (siloxane, Si‒O‒Si), with surface chemistry dominated by silanol (Si‒OH) groups—isolated, geminal, or vicinal. Silanols confer hydrophilicity, weak acidity, and reactivity toward organosilanes [127]. Silanol density and distribution depend on thermal treatment and hydration; dehydroxylation reduces Si‒OH content, increasing siloxane character [128]. In aqueous environments, silanols undergo pH-dependent deprotonation (pKa ~ 4‒7), yielding a negative surface charge above the point of zero charge (~ pH 2‒3) [129]. This combination enables tunable adsorption, colloidal stability, and covalent biofunctionalization [130].

Covalent functionalization of La/Tm-MOFs with an aminated silica shell via siloxane bond formation produces a uniform rod-like core‒shell architecture with a DOX loading capacity of 57.5 mg g⁻¹ over 72 h. The composite exhibits pH-responsive release—negligible at pH 7.4, rapid at pH 3.8‒5.8—due to protonation-induced hydrogen bond disruption. Upon DOX coordination, upconversion fluorescence correlates linearly with cumulative drug release. The system shows low cytotoxicity (> 95% viability) and enables pH-targeted delivery to the acidic TME. The direct proportionality between fluorescence intensity and DOX release permits real-time, non-invasive monitoring without autofluorescence interference. Collectively, this platform integrates pH-responsive release with intrinsic fluorescence tracking, establishing a theranostic strategy to mitigate drug resistance and chemotherapy overtreatment [131].

In additional research, Trushina et al. functionalized UiO‑66 and UiO‑66‑NH₂ MOFs with a mesoporous silica shell (Poly(vinylpyrrolidone) (PVP)) followed by pluronic F127‑FA conjugation via silanol condensation and non‑covalent interactions. The modification reduced surface area, inverted zeta potential from + 39.7 mV to − 33.3 mV, and achieved 5.6 wt% DOX loading; the silica shell limited premature release (12% vs. 3% from uncoated MOFs after 1 h). Biologically, the coating enhanced colloidal stability in cell media (stable 310–330 nm size, low polydispersity), prevented aggregation, and enabled folate‑receptor‑mediated uptake in MCF‑7 cells, with negligible uptake in RAW 264.7 macrophages. Cytotoxicity assays confirmed that DOX‑loaded UiO‑66@SiO₂@F127‑FA NPs exhibited the highest inhibitory effect on MCF‑7 cells, while blank MOFs were non‑toxic, thereby overcoming nonspecific uptake and premature drug release [21].

## Multiple-modifications

In general, single-modifier functionalization of MOFs enables one-step conjugation, induces limited physicochemical changes, and enhances tumor targeting, though performance remains dependent on drug payload. In contrast, multiple modifiers permit sequential surface chemistry, variable zeta potentials, and multifunctionality, providing superior control over drug release, synergistic effects, and reduced premature leakage and systemic toxicity. Consequently, multiple modifiers constitute a multimodal platform capable of overcoming a broader range of biological barriers than single modifiers.

**Scheme 11:**
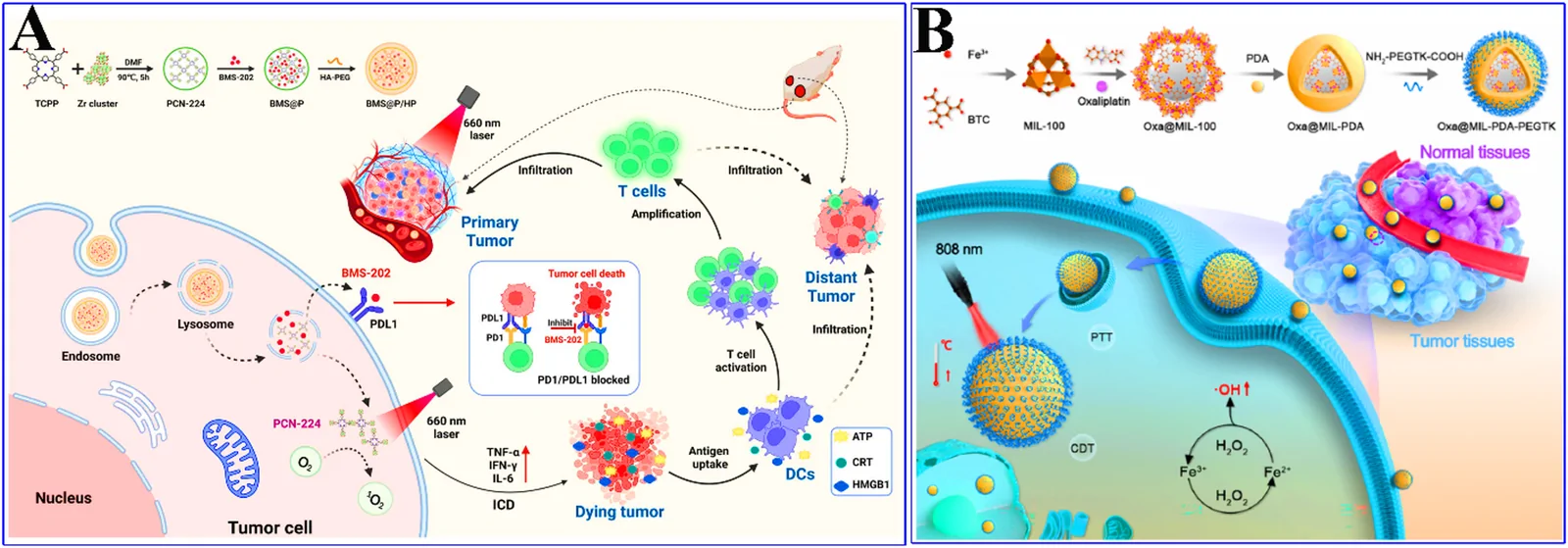
**(A)** Diagram of PDT combined immunotherapy based on MOFs and in vitro release profiles of BMS, BMS@PCN-224 and BMS@P/HP at pH 5.5, 6.5 and 7.4. From ref [132]. with permission. **(B)** schematic representation of Oxa@MIL-PDA-PEGTK NPs’ production and mode of action. From ref [133]. with permission

Accordingly, the covalent conjugation of a HA‑PEG coating onto PCN‑224 via electrostatic and hydrogen‑bonding interactions inverts the zeta potential from + 26.6 mV to − 26.2 mV, yielding uniform 122.8 nm NPs with 25.02% loading of the PD‑L1 inhibitor BMS‑202 and accelerated drug release at pH 5.5 (Scheme 11A**)**. Under 660 nm laser irradiation, the nanocomposite generates singlet oxygen, inducing PDT that triggers immunogenic cell death—evidenced by calreticulin exposure, HMGB1 release, and ATP secretion. This response elevates mature dendritic cells in lymph nodes (1.9‑fold) and intratumoral cytotoxic T lymphocyte infiltration (2.5‑fold), suppressing both primary and distant tumors in a bilateral 4T1 breast cancer model. The HA‑PEG coating further confers low systemic toxicity, overcoming the immunosuppressive TME and enabling effective combinatorial PDT/immunotherapy [132].

In a different work, the nanoplatform is fabricated via sequential PDA coating and covalent conjugation of NH₂‑PEGTK‑COOH onto oxaliplatin (Oxa)‑loaded MIL‑100 (Scheme 11B**)**. The Oxa@MIL‑PDA‑PEGTK exhibits a hydrodynamic diameter of 132.23 nm, an oxaliplatin loading of 5.34 wt%, and reduced surface area and pore volume. Under 808 nm irradiation (100 µg/mL), the NPs achieve a 35 °C temperature rise within 300 s with stable photothermal performance over five cycles and catalyze Fenton‑mediated hydroxyl radical production in a concentration‑dependent manner. In vivo, the NPs preferentially accumulate in tumors, liver, and kidneys; combined with NIR irradiation, they induce robust intratumoral hyperthermia and ROS, leading to complete subcutaneous tumor ablation with minimal weight loss. The platform shows negligible cytotoxicity to normal L02 cells up to 80 µM and avoids the pulmonary congestion and renal edema associated with free oxaliplatin, thereby overcoming off‑target toxicity and improving tumor‑specific delivery in HCC models [133].

Polypeptides confer enhanced receptor affinity and specificity relative to monomeric peptides. A di-peptide formed via dimerization of WQPDTAHHWATL through a lysine residue exhibits strong specific affinity for prostate-specific membrane antigen. In Zhao et al., the Di‑PEG@PTX@ZIF‑8 nanoplatform was surface‑modified by electrostatic adsorption of Di‑PEG‑COOH—synthesized via thiol‒maleimide conjugation between a cysteine‑bearing peptide dimer and Mal‑PEG‑COOH—onto paclitaxel (PTX)‑loaded ZIF‑8. This modification increased particle size (107.4 nm to 126.1 nm), inverted zeta potential (+ 31.5 mV to − 12.3 mV), and altered morphology from sharp dodecahedra to a blunted, film‑covered surface. Drug loading was 12.8% (encapsulation efficiency 55.75%), with minimal release at pH 7.4 and about 60% release within 16 h at pH 5.5. In Lncap cells, Di‑PEG@Cou‑6@ZIF‑8 showed 156‑fold higher uptake than free coumarin‑6 and 1.8‑fold higher uptake than non‑targeted mPEG analogs. Blank Di‑PEG@ZIF‑8 exhibited > 90% viability, whereas Di‑PEG@PTX@ZIF‑8 achieved an IC₅₀ of 0.1052 µg/mL (vs. 9.190 µg/mL for free PTX and 0.1684 µg/mL for mPEG@PTX@ZIF‑8), induced the highest apoptosis rate (56.33%), and inhibited cell migration. Collectively, this platform overcomes poor tumor specificity, premature drug leakage, and insufficient cellular internalization in prostate cancer models [134].

Hydroxyl radicals induce apoptosis via elevated intracellular ROS. Methyl viologen, a redox-active compound, promotes ROS generation through electron transfer [135]. In this context, the nanoplatform is fabricated via layer‑by‑layer electrostatic assembly of polyelectrolyte multilayers (PEM) (polystyrene sulfonate (PSS) and MV‑ polyallylamine hydrochloride (PAH), the latter formed by amide conjugation of methyl viologen‑COOH with PAH) onto DOX‑loaded Fe‑MOF NPs. This modification deposits an about 3 nm coating, increases hydrodynamic size from 102 nm to 110 nm, and induces sequential zeta potential shifts. The platform achieves 88.4% drug loading, remains stable in PBS at 4 °C for four weeks, releases < 4% DOX at pH 7.4 and about 72% at pH 5.0 within 12 h, and exhibits concentration‑dependent Fenton‑mediated hydroxyl radical production. In A549 cells, it enhances cellular uptake, induces ~ 30‑fold higher intracellular ROS than control (8.29‑fold vs. free DOX), achieves > 91% growth inhibition at 40 µg/mL DOX, and yields 62.9% apoptosis (vs. 22.39% for free DOX). This DOX@Fe‑MOF@PEM platform overcomes premature drug leakage, insufficient tumor‑specific release, and inadequate ROS‑mediated cytotoxicity, informing the design of bio‑friendly systems for clinical translation [136]. Such studies can guide the design of future bio-friendly systems that bring us closer to safely translating these platforms into the pharmaceutical consumer market.

## Clinical translation pathway and future perspectives

**Scheme 12:**
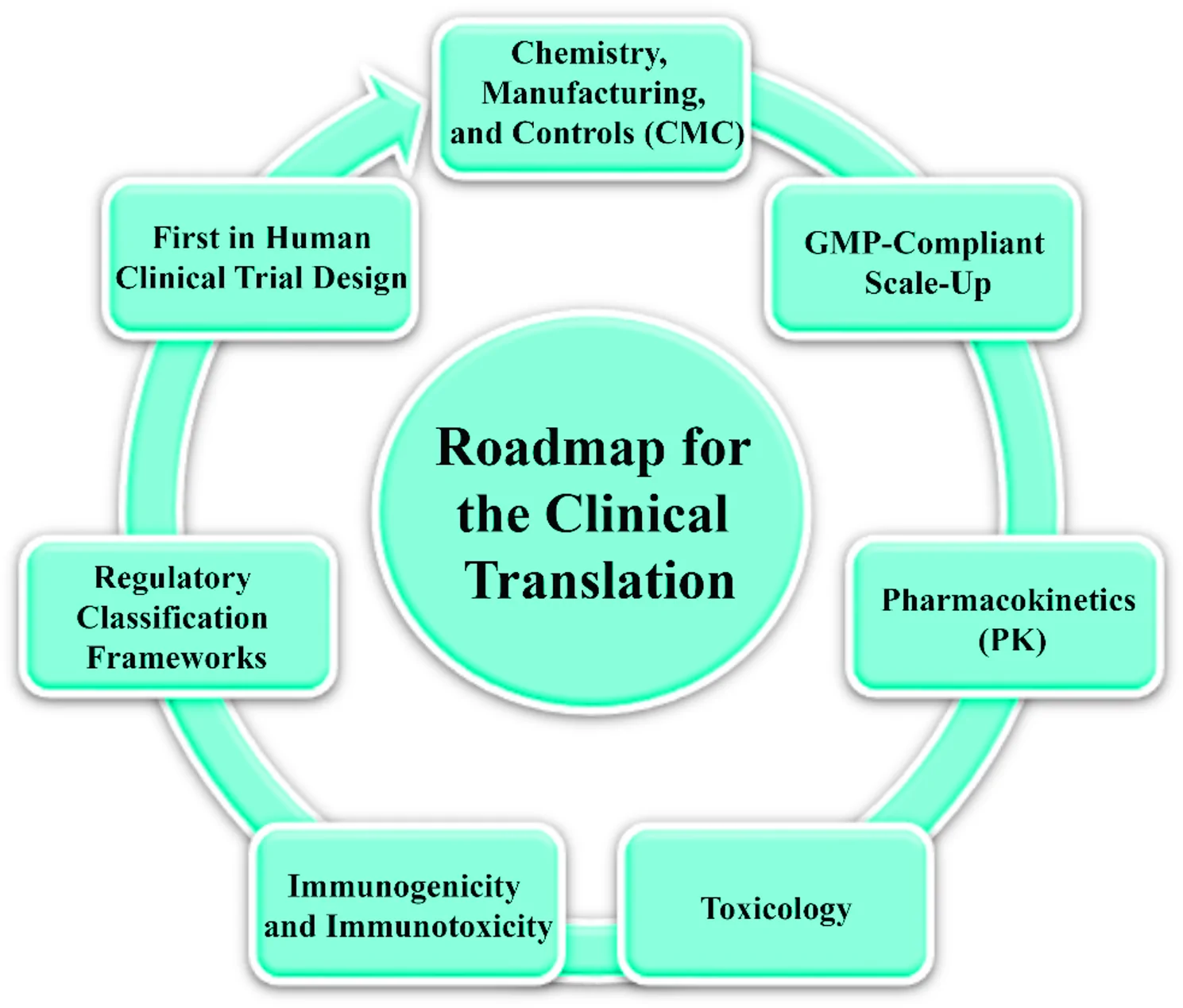
A Comprehensive Roadmap for the Clinical Translation of NMOFs

The path to clinical translation requires a paradigm shift from maximizing therapeutic potency to ensuring predictable, low toxicity biodegradation. Translating a MOF based cancer therapeutic from initial discovery to clinical application and commercial market entry necessitates navigating a series of sequential, rigorously defined steps. Each step is necessitated by specific challenges that must be overcome to ensure structural integrity, reproducibility, manufacturability, and safety [137] **(**Scheme 12**).** The roadmap presents a structured, multidomain pathway for advancing MOF based nanomedicines from laboratory discovery to clinical oncology applications, addressing the critical gap highlighted by the fact that only two MOF candidates have entered human trials to date.

Translating MOFs into approved therapies requires overcoming hurdles in Chemistry, Manufacturing, and Controls (CMC), and regulatory approval. Rigorous structural validation using powder X ray diffraction, BET surface area analysis, thermogravimetric analysis, inductively coupled plasma mass spectrometry, and dynamic light scattering is essential. Critical quality attributes including particle size (50–200 nm), zeta potential, and metal leaching propensity must be defined a priori [96]. Consistent quality across batches is challenged by sensitivity to synthesis parameters, leading to variability in drug loading efficiency and release kinetics. Matching pore size to drug dimensions is critical; alternative loading methods are necessary for oversized drugs [97].

Inter-batch reproducibility remains a significant challenge for MOFs. Minor synthesis variations can produce different phases or properties. For example, an inter-laboratory study of Zr-porphyrin MOFs (PCN-222/Zr [138] and PCN-224/Zr [139]) found that only one of ten laboratories could reproduce phase-pure PCN-222. Similarly, the archetypal Zr-MOF UiO-66 exhibits at least four polymorphic phases accessible under slightly altered conditions. Consequently, “samples of individual MOFs will vary in key physical properties from laboratory to laboratory, making comparison of biological data difficult” [140]. A survey of UiO-66 syntheses in biomedical studies revealed ten different protocols within a single year, yielding widely varying particle sizes, porosities, and surface chemistries [141]. This variability complicates scale-up and quality assurance, as regulators require each batch to meet predefined specifications. Rigorous standardization and full characterization—including size distribution, zeta potential, porosity, defect level, and metal/linker purity—are therefore imperative for every batch. Without these measures, even an established synthesis may produce inconsistent material, undermining reproducibility and safety [141].

Conventional solvothermal or hydrothermal methods are not amenable to scale up; clinical grade production requires microwave assisted synthesis, continuous flow reactors, or ultrasound assisted precipitation. Good Manufacturing Practice (GMP) protocols must address solvent removal per ICH Q3C guideline, endotoxin clearance (≤ 0.5 EU/mg for parenteral products), sterility assurance, and batch release specifications. Scale down qualification and statistical process control are regulatory expectations, with lot to lot consistency in drug loading and release kinetics maintained within a relative standard deviation of ≤ 10% [142].

Intravenously administered MOFs with diameters below 10 nm undergo rapid renal clearance, whereas those exceeding 200 nm are sequestered by the RES, posing a risk of hepatotoxicity. The optimal size range for tumor targeting is 50–150 nm with a neutral or slightly negative surface charge. PEGylation prolongs circulation half life, and active targeting moieties enhance tumor uptake [137]. Comprehensive pharmacokinetics (PK) studies in two species must measure plasma concentration time profiles, tissue distribution, and excretion mass balance, with attention to the fate of both the metal node and the organic linker. A qualified reference standard with batch specific certificates of analysis and stability indicating methods is essential for regulatory submission [137].

According to International Conference on Harmonization (ICH) guidelines, toxicology studies for MOFs require acute (14 day), subchronic (90 day), and for certain MOFs chronic (6 to 9 month) assessments in two species. Required endpoints include clinical observations, body weight and food consumption, hematology, serum chemistry (liver and kidney biomarkers, oxidative stress parameters), histopathology, and genotoxicity [143]. The no observed adverse effect level must be established with a safety margin of at least 10 fold (typically 50 fold) above the projected human equivalent dose. Key toxicity factors include the metal node, organic ligand, surface charge, and particle size. Mitigation strategies comprise polymer coatings, peptide conjugation, biomimetic cell membrane coatings, and biodegradable biomolecular ligands [144]. Immunogenicity assessments must include complement activation, cytokine release, anti drug antibody formation, and evaluation of immune cell populations per ICH S8 guideline. For cancer nanomedicines, some immune activation may be desirable, but uncontrolled inflammation or complement activation related pseudoallergy is unacceptable [145].

Regulatory approval of MOF based cancer nanomedicines requires navigating classification frameworks from the FDA, EMA, and NMPA based on the product’s primary mode of action (PMOA). MOFs delivering a chemotherapeutic agent are regulated as drugs via the FDA CDER NDA pathway. Those conjugated with peptides or antibodies are classified as biologics via FDA CBER. MOFs used solely as contrast agents or physical radioenhancers fall under medical device regulation via FDA CDRH 510(k) or De Novo pathways [146]. Combination products require a formal Request for Designation. Four core characterizations are mandated: particle size distribution using at least two orthogonal techniques; surface chemistry including zeta potential and functional group mapping; aggregation state in biological media under physiologically relevant conditions; and biorelevant drug release at physiological and endosomal pH over clinically relevant timeframes. Compliance is necessary for successful regulatory submission and clinical translation [147, 148].

Currently, anti tumor combined therapy strategies based on MOFs remain distant from clinical application. Efficacy, mechanisms, and preclinical studies require careful evaluation. Following Investigational New Drug (IND) or Clinical Trial Application filing, a Phase I dose escalation study in patients with advanced solid tumors assesses dose limiting toxicities, maximum tolerated dose or recommended Phase II dose, pharmacokinetics, and preliminary anti tumor activity per Response Evaluation Criteria in Solid Tumors [149]. For radioenhancer MOFs, the trial design incorporates radiation fractionation. Phase II focuses on a specific cancer type, often with biomarker driven enrichment. Phase III consists of a randomized controlled trial versus standard of care. Post marketing surveillance and risk evaluation and mitigation strategies are required upon regulatory approval. With a specific focus on MOFs, at present, only two materials have entered human trials: RiMO-401, phase I: NCT06182579; RiMO-301, phase II: NCT0583872910 [150], highlighting a “bench-to-bedside” gap. The Phase I trial of the hafnium‒porphyrin MOF RiMO-301 (NCT03444714) demonstrated excellent safety and early efficacy signals: no dose-limiting toxicities were observed and objective response rates were 38.5% without and 66.7% with PD-1 immunotherapy [150].

## Conclusion and future perspective

Despite advances in cancer biology and the TME, poor prognosis, systemic toxicity, drug resistance, and inadequate targeted therapy persist. MOFs offer high surface area, tunable porosity, and PSM capacity, enabling stimuli‑responsive drug delivery. Polymer, non-polymeric and biomimetic coatings enhance stability and biocompatibility, facilitating combinatorial therapies (e.g., chemotherapy, PDT, catalytic therapy) while reducing off‑target effects. However, each modifier presents limitations; combining multiple modifiers can improve efficacy. Critical challenges remain, including biocompatibility, scalable production, pharmacokinetic optimization, and overcoming biological barriers. Refinements in synthesis and manufacturing are needed to reduce costs. Future regulatory science requires standardized chronic toxicity protocols and routine reporting of long‑term (≥ 6 months) safety data alongside efficacy metrics, after which functionalized MOFs may become translatable oncological tools.

## Electronic Supplementary Material

Below is the link to the electronic supplementary material.

Additional file 1. Table S1

## Data Availability

No datasets were generated or analysed during the current study.

## Abbreviations

MOFs: Metal-organic frameworks
NMOFs: Nanoscale metal-organic frameworks
PSMs: Post-synthetic modifications
MPS: Mononuclear phagocyte system
TME: Tumor microenvironment
NPs: Nanoparticles
ROS: Reactive oxygen species
EPR: Enhanced permeability and retention
GSH: Glutathione
DOX: Doxorubicin
PEG: Polyethylene glycol
PEI: Polyethyleneimine
PAA: Polyacrylic acid
BSA: Bovine serum albumin
CD: Cyclodextrin
HA: Hyaluronic acid
Hep: Heparin
MTX: Methotrexate
ICG: Indocyanine green
PTX: Paclitaxel
PAH: Polyallylamine hydrochloride
PSS: Polystyrene sulfonate
PEM: Polyelectrolyte multilayer
CS: Chitosan
FA: Folic acid
PDT: Photodynamic therapy
PTT: Photothermal therapy
GOx: Glucose oxidase
rGO: Reduced graphene oxide
RBCs: Red blood cells
PDA: Polydopamine
MM: Macrophage membranes
PIP: Piperine
HCC: Hepatocellular carcinoma
PPy: Polypyrrole
PVP: Poly(vinylpyrrolidone)
DDS: Drug delivery system
EM: Erythrocyte membrane
