# Supplementary material for: Functionalized metal-organic frameworks as smart nanoplatforms: advancing tumor precision therapy through post-synthetic surface tailoring, challenges, and prospects for clinical translation
Source: J Nanobiotechnology. 2026 Jul 27;24:980. doi: 10.1186/s12951-026-04757-9 (PMC13689023; doi:10.1186/s12951-026-04757-9)
Supplement: Supplementary file 1 [file 12951_2026_4757_MOESM1_ESM.docx]

**Supplementary Table S1:** An overview of PSMs in MOF‑based systems and their tumor therapy applications.

| Type of the MOF | Clas. | The modifier | MOF cargo | Size | Treatment /in vitro or in vivo | Conclusions | Ref. |
| --- | --- | --- | --- | --- | --- | --- | --- |
| PCN-224 | Natural polymer | HA | DOX | ∼250± 20 nm | RPMI 1640 cells-in vitro | 1. Enhance hydrophilicity, biocompatibility, biodegradability, cell uptake, and colloidal stability, 2. CD44‑mediated active targeting, 3. HAase-responsiveness, 4.prolonged circulation time in vivo | (75) |
|  | Synthetic polymer | PAH | Indocyanine green (ICG) | 112 nm | MDA-MB-231-in vivo | PAH acts as an interfacial linker or electrostatic glue that facilitates the stable attachment of ICG (Surface Charge Reversal) | (151) |
|  | Non-polymeric | Biomimetic camouflage | Gox,  L-Arg | 129 nm | 4 T1- in vivo | 1.Superior homologous targeting, 2. immune escape, 3. selective uptake in 4T1 cells over HepG2 cells | (111) |
| UIO-67 | Natural polymer | streptavidin | - | 231.91 ± 16.22 nm | HeLa and MCF-7 - in vitro | facilitates the attachment of biotinylated antibodies, 2. enhances the functionalization of the MOF surface, allowing for the capture and subsequent release | (152) |
| UiO-66 | Synthetic polymer | PEI | plasmid (pEGFP) | 240.6 ± 44.4 nm | A549- in vitro | 1. Protect the nucleotides from degradation,2. provide endosomal escape. | (100) |
|  | Non-polymeric | SiO2-FA | DOX | 163±53 nm | MCF-7- in vivo | 1.Improved Colloidal Stability, 2. controlled Drug Release, 3.grafted a pluronic F127-FA conjugate onto the silica surface to achieve active targeting of folate | (21) |
|  | Synthetic polymer | PEG | AQ4N | 95 nm | U87MG /in vivo | 1. Improve colloidal stability, 2. Enable “stealth” properties, 3. prevent protein adsorption | (153) |
| ZIF-8 | Non-polymeric | Liposome | naringenin (NAR) | 113.2 ± 1.4 nm | A549, SGC-7901 -in vitro | 1. enhance the drug's bioavailability; 2. boost its sustained-release features; 3. lessen adverse effects; and 4. elevate its effectiveness. | (16) |
|  | Multiple-modification | PDA + HA | DOX | 150 nm | prostate cancer in vitro | 1. sustained and pH-sensitive release; 2. Active tumor targeting; 3. Enhanced cellular uptake ; and 4. Fe^3+^‑PDA enables T₁‑weighted MR imaging. | (154) |
|  | Multiple-modification | CS + methylcellulose | DOX | 250 nm | 4T1, L929 - in vivo | 1. Injectable and self‑healing, 2. Controlled and sustained release, 3. Biocompatible and biodegradable, 4. Local “drug store”preventing their rapid clearance and reducing systemic toxicity. | (155) |
|  | Multiple-modification | silica + PEG | DOX/DNA/mRNA/RNP/RNP + ssODN | 110 nm | HCT 116 -in vivo | 1. enhance biocompatibility and physical stability, 2. Prevent particles from agglomeration | (156) |
| MIL-100 | Multiple-modification | β-CD+ mannose + TEG | DOX | 224 - 21 nm | J774A.1-in vitro | 1. CD=Improves stability, prevents pore penetration, 2. Mannose =Increase cell uptake due to targets lectin receptors, 3. TEG ‒ for “stealth” properties and reduces protein adsorption and macrophage clearance | (157) |
|  | Multiple-modification | (PEG+p(Asp-Dopa) | Pd^2+^ | 170±7 to 153 ± 6 nm | B16F10, C57BL/6 -in vivo | 1. Improve colloidal stability, 2. Prolong blood circulation time, 3. Enhance tumor accumulation | (32) |
|  | Synthetic polymer | PEG | DOX and ICG | 150 nm | 4 T1 and MCF-7 /In vivo | 1. Improve colloidal stability, 2. Reduce non‑specific interactions | (158) |
| Fe-soc-MOF | Multiple-modification | PEG + PPy | - | Fe-soc-MOF < 100 nm | 4T1 -in vivo | 1. Convert hydrophobic MOF to water‑dispersible, 2. Improve biocompatibility, 3. PPy has strong absorption in the near‑infrared (NIR) region (808 nm) and enable PTT | (159) |
| MIL-Fe | Synthetic polymer | PVP | DOX | ~300 nm | CAL27 and NIH/3T3/ in vivo | 1. Improve biocompatibility, 2.reduce cytotoxicity, 3.enhance colloidal stability, 4.enable safe in vivo application | (160) |
| MIL‑125 (Ti) | Multiple-modification | poly (aniline-co-para phenylenediamine) | DOX/pCRISPR | - | MCF-7, HEK-293, NIH3T3 - in vitro | 1. Improved stability and biocompatibility, 2. Improve cellular uptake, 3.facilitates endosomal escape, 4. Multimodal theranostic functionality | (161) |
| Mn-SS MOF | Multiple-modification | PDA + PEG | DOX | ∼100 nm | Breast cancer in vivo | 1. Prevent NPs aggregation, 2. Enhance physiological stability and biocompatibility, 3. Prolong blood circulation time | (162) |
| Eu-MOF | Synthetic polymer | PEG | Apatinib | 365 nm | HepG2 /in vivo | 1. Enhances biocompatibility, 2. Prolongs blood circulation | (163) |
| Gd‑MOF and Yb‑MOF | Natural polymer | D-glucose | DOX | 182 nm | GLUT1 positive cell line HeLa, HUVEC-in vivo | 1. Improve biocompatibility, 2. Enable active tumor target the glucose transporter protein 1 (GLUT1), 3. controls drug release, 4. Act as a pH‑responsive “gatekeeper” | (164) |
